# Ambient Temperature is A Strong Selective Factor Influencing Human Development and Immunity

**DOI:** 10.1016/j.gpb.2019.11.009

**Published:** 2020-08-19

**Authors:** Lindan Ji, Dongdong Wu, Haibing Xie, Binbin Yao, Yanming Chen, David M. Irwin, Dan Huang, Jin Xu, Nelson L.S. Tang, Yaping Zhang

**Affiliations:** 1 State Key Laboratory of Genetic Resources and Evolution, Yunnan Laboratory of Molecular Biology of Domestic Animals, Kunming Institute of Zoology, Chinese Academy of Sciences, Kunming 650223, China; 2 Department of Biochemistry, Medical School of Ningbo University, Ningbo 315211, China; 3 Department of Preventive Medicine, Medical School of Ningbo University, Ningbo 315211, China; 4 Department of Laboratory Medicine and Pathobiology, University of Toronto, Toronto, ON M5S 1A8, Canada; 5 Banting and Best Diabetes Centre, University of Toronto, Toronto, ON M5S 1A8, Canada; 6 Department of Chemical Pathology, and Laboratory for Genetics of Disease Susceptibility, Li Ka Shing Institute of Health Sciences, Faculty of Medicine, The Chinese University of Hong Kong, Hong Kong Special Administrative Region, China; 7 KIZ/CUHK Joint Laboratory of Bioresources and Molecular Research in Common Diseases, Kunming 650223, China; 8 Laboratory for Conservation and Utilization of Bio-resource, Yunnan University, Kunming 650091, China

**Keywords:** Solar radiation, Ambient temperature, Natural selection, Development, Immunity

## Abstract

Solar energy, which is essential for the origin and evolution of all life forms on Earth, can be objectively recorded through attributes such as climatic **ambient temperature** (CAT), ultraviolet radiation (UVR), and sunlight duration (SD). These attributes have specific geographical variations and may cause different adaptation traits. However, the adaptation profile of each attribute and the selective role of solar energy as a whole during human evolution remain elusive. Here, we performed a genome-wide adaptation study with respect to CAT, UVR, and SD using the Human Genome Diversity Project-Centre Etude Polymorphism Humain (HGDP-CEPH) panel data. We singled out CAT as the most important driving force with the highest number of adaptive loci (6 SNPs at the genome-wide 1 × 10^−7^ level; 401 at the suggestive 1 × 10^−5^ level). Five of the six genome-wide significant adaptation SNPs were successfully replicated in an independent Chinese population (*N* = 1395). The corresponding 316 CAT adaptation genes were mostly involved in **development** and **immunity**. In addition, 265 (84%) genes were related to at least one genome-wide association study (GWAS)-mapped human trait, being significantly enriched in anthropometric loci such as those associated with body mass index (*χ*^2^; *P* < 0.005), immunity, metabolic syndrome, and cancer (*χ*^2^; *P* < 0.05). For these adaptive SNPs, balancing selection was evident in Euro-Asians, whereas obvious positive and/or purifying selection was observed in Africans. Taken together, our study indicates that CAT is the most important attribute of solar energy that has driven genetic adaptation in development and immunity among global human populations. It also supports the non-neutral hypothesis for the origin of disease-predisposition alleles in common diseases.

## Introduction

Solar radiation is an essential environmental factor in almost all ecosystems, and it is fundamental to the survival of the majority of life forms, including human beings [1]. Three attributes of solar radiation, including light intensity (ultraviolet radiation, UVR), climatic ambient temperature (CAT), and photoperiod (day/night cycle indicated by sunshine duration, SD), have been identified as important components of geographic variation in solar radiation exposure [2]. Together, these three attributes have shaped and characterized distinct habitats in which specific adaptive mechanisms could occur.

Physiological studies have been intensively carried out to investigate the morphological adaptation to solar radiation in humans. Sunlight intensity is the most commonly researched, and it is found to correlate well with the worldwide variation in human skin pigmentation [3]. Proposed driving mechanisms include photosynthesis of vitamin D [4] associated with ultraviolet B (UVB), photolysis of folate [5] with UVA, or a combination of the two. In addition, overexposure to sunlight, particularly UVB, is a recently well-recognized etiologic agent for skin cancer, which suggests a candidate protective mechanism of melanin [6]. CAT has a close association with the morphology of the human body, including body size, limb proportion, and pelvis form [7], [8], consistent with Bergmann’s and Allen’s rules [9], [10]. That is, in wide-ranging homeotherms, larger body size and shorter appendages will be found in the colder parts of the range, and vice versa. Furthermore, both elevated basal metabolic rates in arctic populations as a local adaptation to cold stress [11] and short stature in African and American tropical forest residents due to inefficient thermoregulation [12] suggest a thermal adaptation mechanism in response to CAT. By contrast, daily fluctuations and seasonal changes in these three attributes, especially in the photoperiod as indicated by SD, help entrain biological rhythms, which are of fundamental importance for human immunity and physiology [13], [14], [15]. Circadian misalignment involved in shift work has been implicated to increase the risk of metabolic syndrome, immune disorders, and many other diseases [16]. Therefore, a biological rhythm adaptation in response to SD is also strongly suggested as a human adaptation mechanism to solar radiation.

Also, genetic studies have been increasingly used to help elucidate the mechanisms of human physiological adaptation to solar radiation. To date, genes including *ASIP*, *ATRN*, *DCT*, *HERC2*, *IRF4*, *MC1R*, *OCA2*, *SLC24A5*, *SLC45A2*, *TYR*, and *TYRP1* have been identified to correlate with skin color [17]. However, these genes were revealed mainly through candidate genetic divergence studies between dark- and light-skinned populations. Recently, identification of sun-exposure-dependent gene expression quantitative trait loci (eQTLs) in *SLC45A2* confirmed human adaptation to local variant levels of sunlight [18]. To cope with the harsh tropical forest environment, Africans and Amerindians may follow different routes through convergent genome-wide signals related to lipid metabolism, the immune system, body development, and RNA polymerase III transcription initiation [12]. By contrast, local adaptation to cold stress and a high marine-derived fat diet in a Greenlandic Inuit population led to the selection of the *FADS* region, which encodes fatty acid desaturases [19]. Interestingly, the cold-responsive *FADS* region is closely associated with human body height and weight [19]. However, only a few genes related to biological rhythms have been identified under selection. Among them, genetic polymorphisms of the *PER2* gene demonstrate a significant geographic distribution, which is suggestive of local directional selection [20]. Our previous work examined the melatonin pathway and found that the *MTNR1B* gene encoding melatonin receptor 2 has undergone the natural selection of population residential SD [15].

Although several aforementioned adaptive mechanisms have been suggested for single attribute—photosynthesis of vitamin D and/or photolysis of folate together with recently suggested protective mechanism of melanin for UVR; a thermal adaptation mechanism in response to CAT; and a biological rhythm adaptation in response to SD—none of the present studies has investigated the three attributes of solar radiation simultaneously. Thus far, the current physiological and genetic data are still insufficient to elucidate the overall selective role of solar radiation as an integrative environmental niche. In particular, it is unknown (1) which attribute of solar radiation played the dominant role, (2) which category of genes adapted most to these environmental attributes, and (3) what the underlying selection mechanism was.

To answer these questions, we conducted a series of analyses using an extensive genetic dataset generated from 40 subpopulations in the worldwide Human Genome Diversity Project-Centre Etude Polymorphism Humain (HGDP-CEPH) panel (*N* = 764) [21]. First, through a series of genome-wide adaptation studies (GWAdS), we investigated which physical attribute of solar radiation, *i.e.*, CAT, SD, or UVR, has the strongest selective effect on human genome diversity. Genome-wide significant GWAdS SNPs were replicated by genotyping a cohort of Chinese individuals sampled from broad geographic ranges and by *in-silico* online analysis of dbCLINE database [22]. Next, we further characterized categories of genes showing adaptation to these attributes through Gene Ontology (GO) annotation [23] and Kyoto Encyclopedia of Genes and Genomes (KEGG) pathway analysis [24]. Additionally, putative GWAdS SNPs were mapped against the GWAS Catalog [25] to explore potential association with known traits. Their effects on gene regulation and transcriptional regulation were retrieved from Genotype-Tissue Expression (GTEx) and HaploReg [26], [27]. Finally, we examined the underlying selection mechanisms through analyses of derived allele frequencies (DAFs) [28], Wright’s fixation index (*F*_ST_) [29], and integrated haplotype scores (iHSs) [30]. Collectively, the results of this study suggest that solar radiation may have a selective role in human development and immunity genes mainly through CAT.

## Results

### Results of the GWAdS

**Figure 1:**
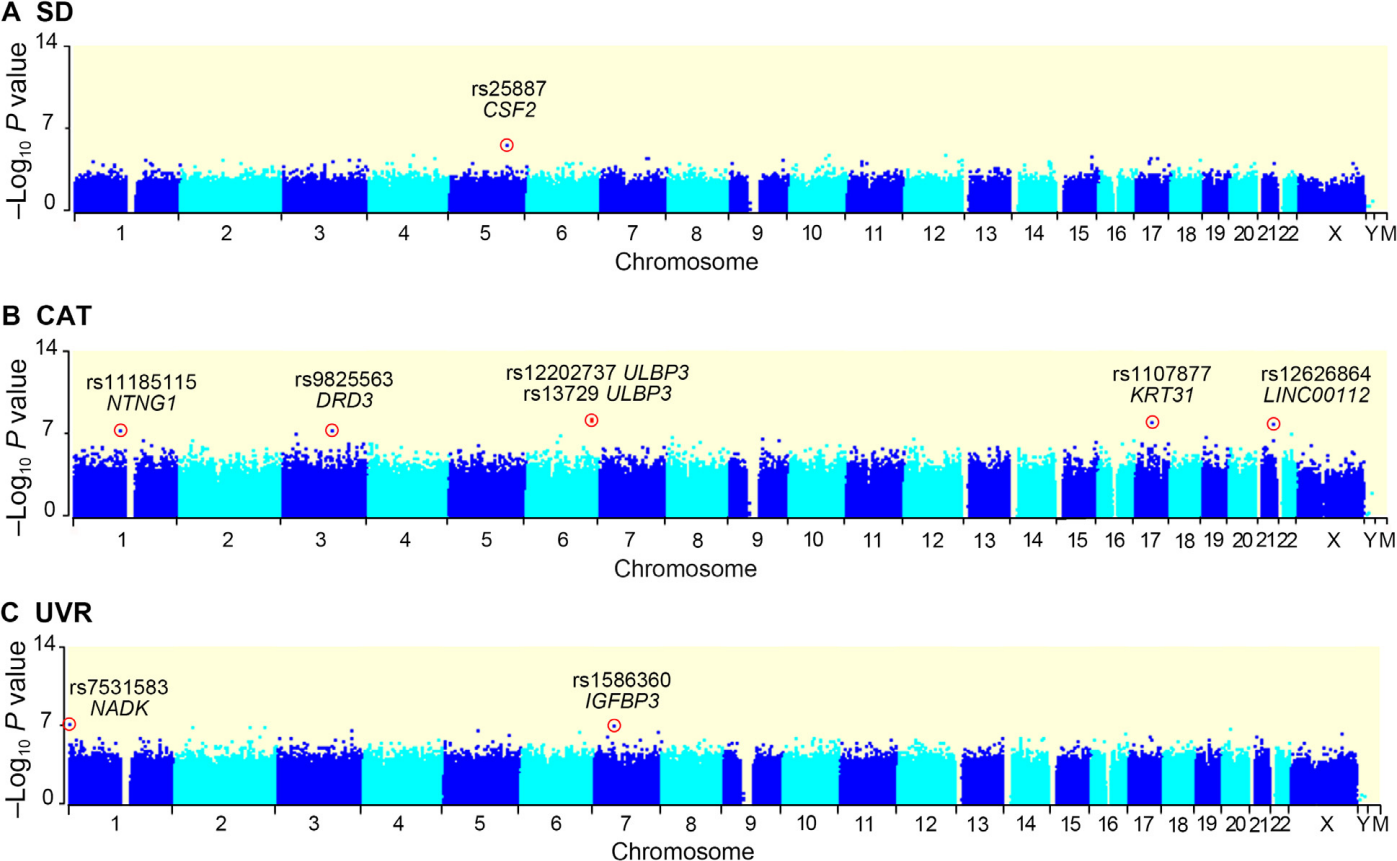
**Manhattan plots showing the significance of associationof all 516,663 SNPs with CAT, SD, and UVR** SNPs are plotted on the x-axis according to their position on each chromosome; associations with the three environmental factors are indicated on the y-axis (as –log_10_*P* value). Six CAT-associated SNPs (*P* ≤ 1 × 10^−7^), one SD-associated SNP (*P* ≤ 1 × 10^−5^) and two UVR-associated SNPs (*P* ≤ 1 × 10^−7^) are indicated with circles. CAT, climatic ambient temperature; SD, sunlight duration; UVR, ultraviolet radiation.

For the 516,663 common SNPs, after Pearson bivariate correlation and multiple linear regression analysis, CAT had a greater number of significantly associated SNPs (six SNPs at the genome-wide 1 × 10^−7^ level; a total of 401 SNPs at the suggestive 1 × 10^−5^ level) than did UVR (two genome-wide SNPs; 233 suggestive SNPs) and SD (one suggestive SNP) (Figure 1). The six genome-wide CAT-associated SNPs were in or near five genes, UL16 binding protein 3 (*ULBP3*), keratin 31 (*KRT31*), long intergenic non-protein coding RNA 112 (*LINC00112*), dopamine receptor D3 (*DRD3*), and netrin G1 (*NTNG1*), with two hits at *ULBP3*. The two significant UVR SNPs were linked to NAD kinase (*NADK*) and insulin-like growth factor binding protein 3 (*IGFBP3*). All these SNPs were still significant after false discovery rate (FDR) corrections (modified positive false discovery rate, pFDR; *P* < 0.01, Tables S1–S3).

### Replication of the genome-wide adaptation signals in Chinese populations

After using a haplotype tagging technique and a minor allele frequency (MAF) filter, five genome-wide significant CAT-associated SNPs and one UVR SNP were experimentally validated in 1395 Chinese individuals. Neither significant deviation from Hardy-Weinberg equilibrium (HWE) nor gender or age difference in allele frequencies was observed (*P* > 0.05).

**Supplementary Figure S1:**
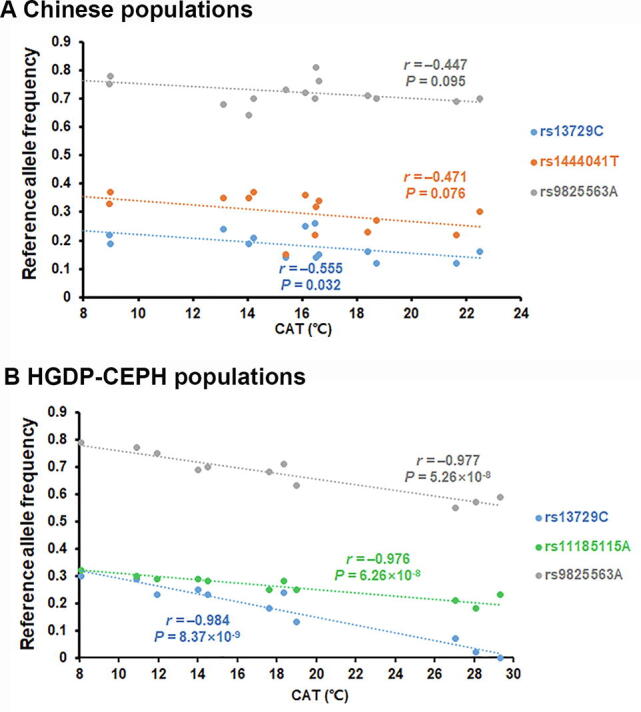
**Schematic representation of linear correlation for three CAT-associated SNPs in Chinese (A)and worldwide HGDP-CEPH(B)populations** In Chinese populations, rs11185115A within *NTNG1* was replaced by nearby rs1444041T (*r*^2^ = 0.954), which was not in the original dataset, owing to a lack of the appropriate SNPstream primers.

Among the five replicated CAT-associated SNPs, the rs13729C allele frequency in *ULBP3* was negatively associated with the annual average CAT (*r* = –0.555, *P* = 0.032), whereas rs1444041T in *NTNG1* (representing rs11185115A, *r^2^* = 0.954) and rs9825563A in *DRD3* had marginally negative associations (Figure S1A; Table S4). They did show consistent trends as observed in the HGDP-CEPH populations (Figure S1B; Table S5); that is, all three DAFs increase with declining annual average CAT. When additional temperature indexes were examined, all five SNPs, except rs1107877 in *KRT31*, were all significantly associated with annual average CAT range (Pearson bivariate correlation; *P* < 0.05). For these four SNPs, rs12626864 in *LINC00112* and rs13729 in *ULBP3* were associated with an annual average maximum CAT and annual average minimum CAT, respectively. In addition, except rs12626864, the remaining three SNPs were associated with annual average extremely minimum CAT (Table 1**).** However, no SNP was significantly associated with annual average extremely maximum CAT. On the other hand, the six genome-wide CAT SNPs, except rs9825563 in *DRD3* being associated with intensive agriculture, were confirmed to be all significantly associated with minimum temperature (winter) in dbCLINE database (Table 2), which examined 61 worldwide populations including the 52 HGDP-CEPH populations using a different transformed rank statistic method [22].

**Table 1:** **Replication study for five CAT-associatedSNPs in Chinese populations**

*Note*: Significant associations between SNPs and CAT variables are indicated using √ (Pearson bivariate correlation; *P* < 0.05). rs12202737 is in complete linkage disequilibrium with rs13729 (*r^2^* = 1) in HapMap CHB population. rs11185115 within *NTNG1* is tagged by SNP rs1444041 (*r^2^* = 0.954) due to lack of appropriate SNPstream primers. $\bar{T}$ means annual average CAT. CAT, climatic ambient temperature (℃).

**Table 2:** **Replication study for six CAT-associatedSNPs in dbCLINE**

For UVR-associated rs7531583 in *NADK*, the G allele frequency was associated with summer UVR dose (June, July, and August, *P* < 0.05) instead of annual average total UVR dose. However, the two UVR SNPs failed to be replicated in the dbCLINE. Only rs7531583 was found to be associated with absolute latitude (transformed rank statistic 0.002).

### Potential functional implications of the genome-wide significant signals

*In-silico* analysis was used to annotate these SNPs in the GTEx project [26] and HaploReg database [27] for known tissue eQTLs and functional noncoding genomic regions, respectively (Tables S6 and S7). Five out of six CAT (except rs11185115 of *NTNG1*) and both of the UVR-associated signals might have functional regulation on transcription.

Five out of the six CAT signals (except rs11185115 from *NTNG1* gene) are predicted to affect known transcription factor regulatory motifs: rs12202737 and rs13729 are putative *cis*-eQTLs for *ULBP3* and *RAET1L*, while rs1107877 of *KRT31* acts as a *cis*-eQTL for *KRT32* and *KRTAP3-2*. For the two UVR signals, rs7531583 of *NADK* could *cis*-regulate itself and five nearby genes including *CALML6*, *CDK11A*, *MMP23A*, *RP1-140A9.1*, and *TMEM52*; rs1586360 from *IGFBP3* could regulate *IGFBP1*. In addition, they both were predicted to affect transcription factor motifs for a given proximal gene.

### Categories of genes with significant solar radiation signals

**Supplementary Figure S2:**
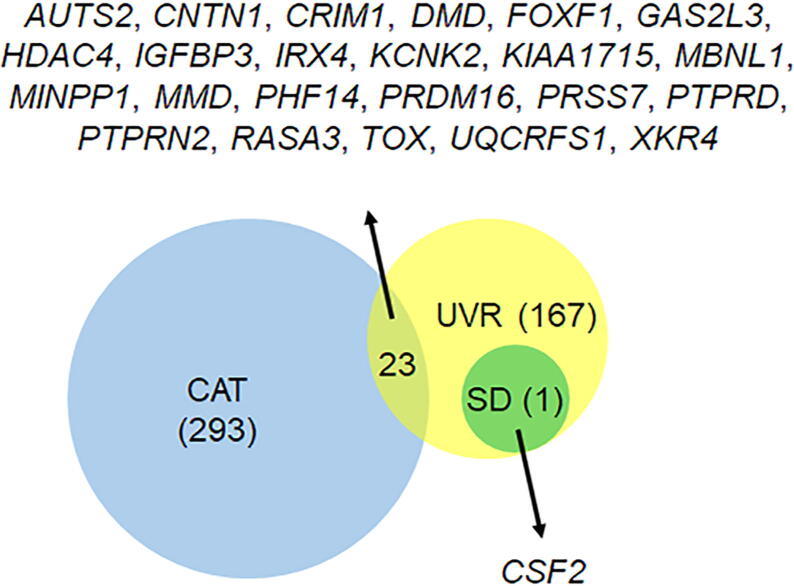
**Overlapping genes among CAT, SD, and UVR (*P* ≤ 1 × 10^−5^)** CAT, climatic ambient temperature; UVR, ultraviolet radiation; SD, sunshine duration.

Although only 8 SNPs reached genome-wide significance, 629 SNPs are putatively adaptive (*P* ≤ 1 × 10^−5^). At this level, 316 CAT-associated genes, 191 UVR-associated genes, and a single SD-associated gene were identified. Among them, the SD-associated *CSF2* was also targeted by UVR, while 23 other UVR-associated genes, including the genome-wide significant *IGFBP3*, were also targeted by CAT (Figure S2). Overall, a total of 484 non-redundant genes were mapped to GO annotations and KEGG pathways. In comparison with the null distribution, no significant KEGG pathway was observed, but GO terms involving developmental processes and immunity were significantly enriched (*χ^2^*; *P* < 5 × 10^−4^, Table S8).

When examined separately, only the 316 CAT-associated genes showed a significant clustering with developmental processes and immunity after genomic control (Table 3). More importantly, the adjusted fold-enrichment increased significantly compared with the data obtained using the full 484 genes, with a newly emerging GO category: 048513 organ development. Specially, the fold enrichment is significantly higher (*χ^2^*; *P* < 5 × 10^−9^) in categories for immunoglobulin I-set domain (5.03), immunoglobulin C2-set domain (4.33), regulation of cell differentiation (2.58), and regulation of developmental process (2.14). To this end, we focused on the CAT-associated signals with respect to potential GWAS traits and evolutionary analyses to examine the underlying selective mechanisms.

**Table 3:** **GO annotations for CAT-associatedgenes and significant categories**

*Note*: Bonferroni *P* values are calculated from the modified one-tail Fisher Exact Test, supplied by the DAVID online analysis. Percentage of related genes is the percentage of each category genes found among all CAT-associated genes. Adjusted fold enrichment derives from the ratio of the percentages for each category genes found among CAT-associated genes and all 17,169 genes from the Illumina 650Y platform. Similarly, *χ^2^ P* values are calculated according to the numbers of related genes for CAT-associated genes and for the 17,169 genes. Here, number and percentage of each GO category for all 17,169 Illumina 650Y platform genes work as null distributions.

### GWAS mapped traits or human diseases

Owing to the various linkage disequilibrium patterns revealed by different sets of tagging SNPs among human populations, we only examined potential phenotype effect for the 316 CAT-associated genes in the GWAS Catalog. Among them, 265 genes were associated with a GWAS trait or human disease. To be more specific, 102 genes were associated with human anthropometric traits including height (21), weight (5), obesity (46), waist-to-hip ratio (8), fat (7), adipose (11), and body mass index (BMI, 32), showing significant enrichment compared with the two null distributions of all GWAS genes and HGDP-CEPH genes (*χ^2^*; *P* < 0.001). When examined separately, waist-hip ratio and obesity (early onset extreme) showed the most significant enrichment (*χ^2^*; *P* < 0.005, Table S9).

Interestingly, other GWAS traits including immunity, cancer, mental function, and mental disorders also showed significant enrichment (*χ^2^*; *P* < 0.05, Table S9). Here, immunity associated traits mainly include acne (severe), parasitemia in *Trypanosoma cruzi* seropositivity, inflammatory biomarkers, allergic rhinitis, immune response to smallpox (secreted IFN-alpha), diisocyanate-induced asthma, inflammatory skin, and bowel diseases. For cancers, the main types involved are prostate cancer and colorectal cancer. For mental disorders, the main related types are referred to eating disorders (purging via substances), alcohol dependence, bipolar disorder and schizophrenia, major depressive disorder, schizophrenia, *etc.*

### Evolutionary analyses of the CAT-associated SNPs

Among the 401 suggestive SNPs, 49 (46 ancestral alleles) were completely fixed in all four examined African populations, with additional SNPs partially fixed in at least one African population. All these SNPs were polymorphic in the Asian, European, and Middle Eastern populations, with the exception of Siberians (Table S10).

**Figure 2:**
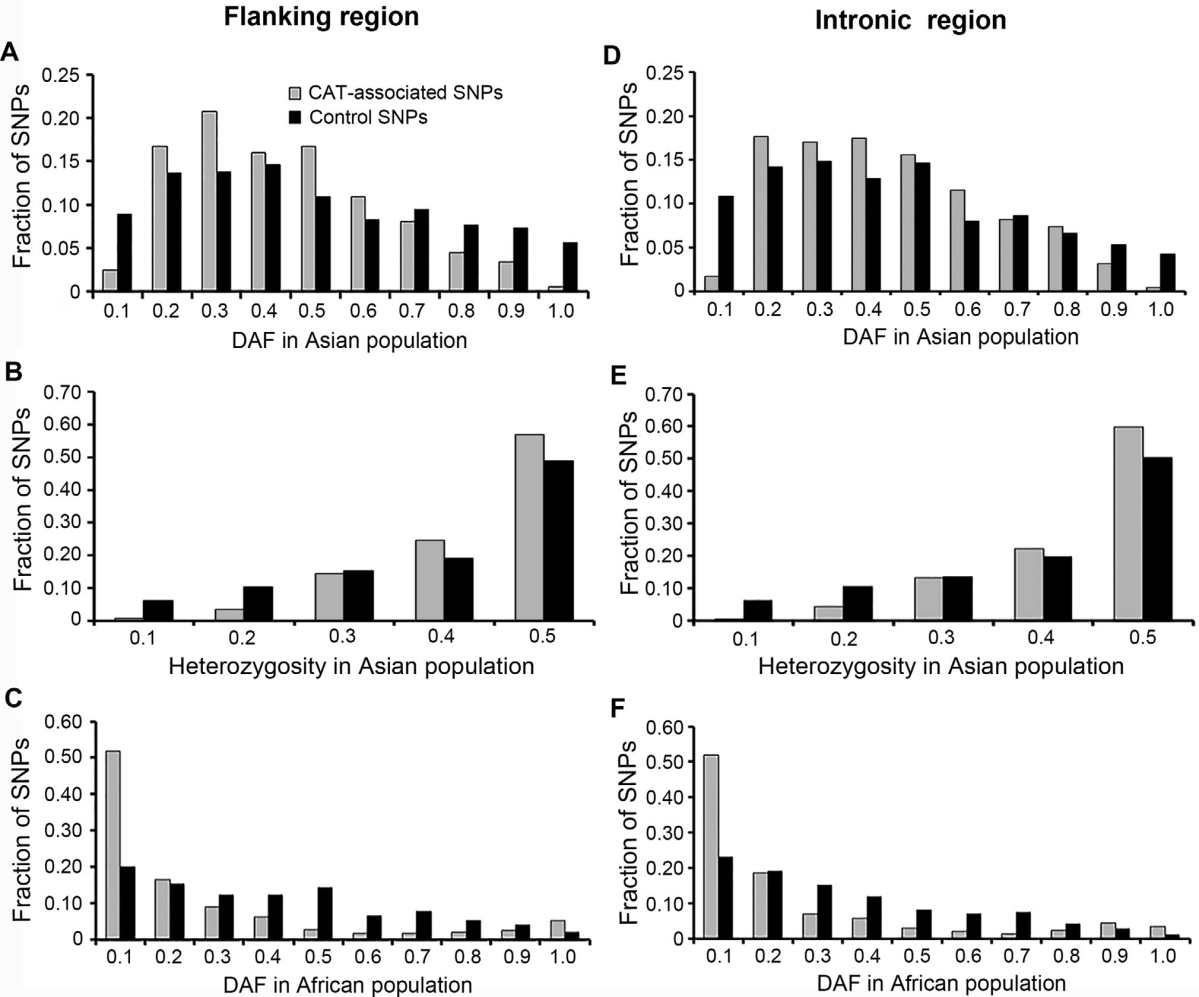
**DAF spectrums of CAT-associated flanking regionand intronic region SNPs in Asian and African populations** DAF distributions of flanking region SNPs in Asian (**A**) and African populations (**C**). DAF distributions of intronic region SNPs in Asian (**D**) and African populations (**F**). Heterozygosity plots of Asian population for flanking region SNPs (**B**) and intronic region SNPs (**E**). DAF, derived allele frequency.

**Supplementary Figure S3:**
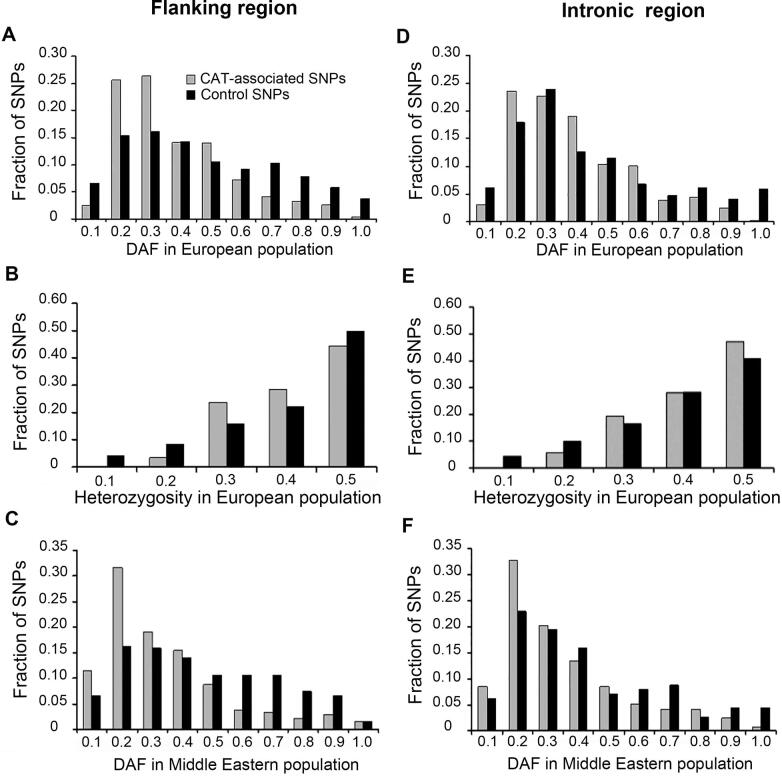
**DAF spectrums of CAT-associated SNPs in European and Middle Eastern populations** DAF distributions of flanking region SNPs in European **(A)** and Middle Eastern populations **(C)**. DAF distributions of intronic region SNPs in European **(D)** and Middle Eastern populations **(F)**. Heterozygosity plots of European population for flanking region SNPs **(B)** and intronic region SNPs **(E)**. DAF, derived allele frequency.

**Supplementary Figure S4:**
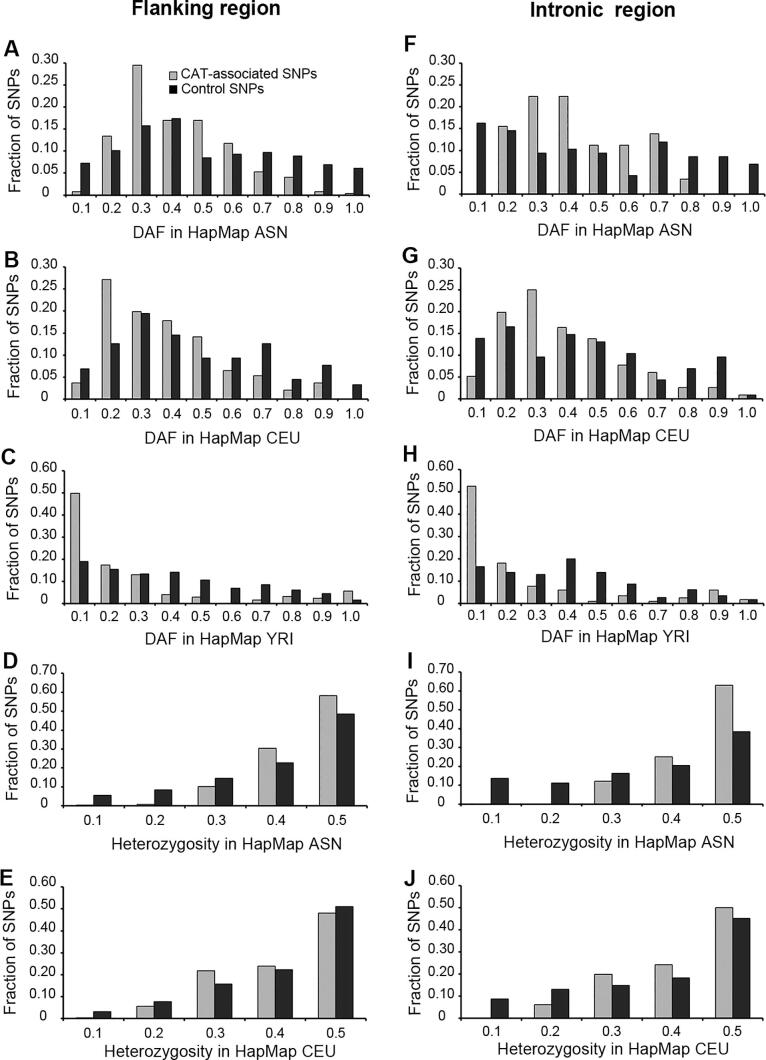
**DAF spectrums of CAT-associated SNPs in HapMap populations** DAF distributions of flanking region SNPs in ASN **(A)**, CEU **(B)**, and YRI **(C)**. DAF distributions of intronic region SNPs in ASN **(F)**, CEU **(G)**, and YRI **(H)**. Heterozygosity plots of ASN for flanking region SNPs **(D)** and intronic region SNPs **(I)**. Heterozygosity plots of CEU for flanking region SNPs **(E)** and intronic region SNPs **(J)**. DAF, derived allele frequency. ASN, Asians, includes CHB and JPT. CEU, Caucasians. YRI, Africans.

The DAF spectrum of the CAT-associated SNPs in four continental populations, Asian, European, Middle Eastern, and Sub-Saharan African populations merged from HGDP-CEPH populations (Table S11), was first analyzed. In Asians, the DAF distribution skewed toward intermediate-frequency derived alleles (2*pq* > 0.40, *P* < 0.01), with a significant scarcity of rare derived alleles (DAF ≤ 0.10, *P* < 5 × 10^−8^) and high-frequency derived alleles (DAF > 0.90, *P* < 0.01) relative to control SNPs (Figure 2). By contrast, the DAF distribution in the African populations, especially for the three tropical populations (Kenya, Nigeria, and Senegal), skewed toward rare derived alleles (*P* < 1 × 10^−16^, Figure 2). For Europeans, a non-significant excess of intermediate-frequency derived alleles was also observed while no specific DAF distribution pattern was observed in Middle Eastern populations (Figure S3). In the HapMap populations, we observed similar trends as the HGDP-CEPH populations for Asians (ASN, comprised of CHB and JPT) and Africans (YRI), with a weaker statistical significance (*P* < 0.01, Figure S4). On the contrary, Caucasians (CEU) as Europeans showed a statistical significance (*P* < 0.01). Taken together, DAF distribution patterns propose balancing selection as the major evolutionary driver for both Asians and Europeans, and in Africans if considered the large proportion of fixed SNPs positive (complete sweeps) or purifying selection was suggested.

**Supplementary Figure S5:**
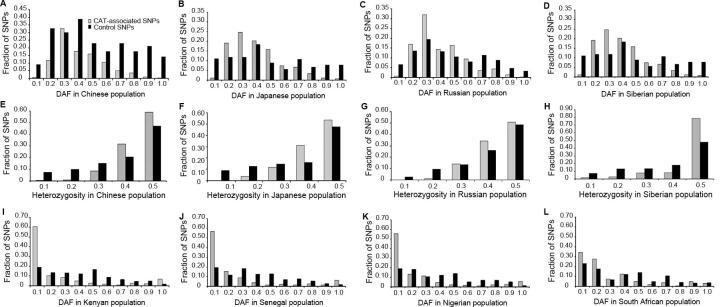
**DAF spectrums of CAT-associated SNPs in Chinese, Japanese, Russian, Siberian, and four African populations (flanking region SNPs)** DAF distributions of flanking region SNPs in Chinese **(A)**, Japanese **(B)**, Russian **(C)**, Siberian **(D)**, Kenyan **(I)**, Senegal **(J)**, Nigerian **(K)**, and South African populations **(L)**. Heterozygosity plots of flanking region SNPs in Chinese **(E)**, Japanese **(F)**, Russian **(G)**, and Siberian populations **(H)**. DAF, derived allele frequency.

**Supplementary Figure S6:**
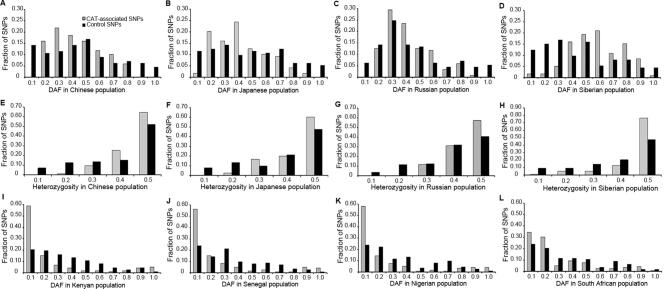
**DAF spectrums of CAT-associated SNPs in Chinese, Japanese, Russian, Siberian, and four African populations (intronic SNPs)** DAF distributions of intronic region SNPs in Chinese **(A)**, Japanese **(B)**, Russian **(C)**, Siberian **(D)**, Kenyan **(I)**, Senegal **(J)**, Nigerian **(K)**, and South African populations **(L)**. Heterozygosity plots of intronic region SNPs in Chinese **(E)**, Japanese **(F)**, Russian **(G)**, and Siberian populations **(H)**. DAF, derived allele frequency.

**Supplementary Figure S7:**
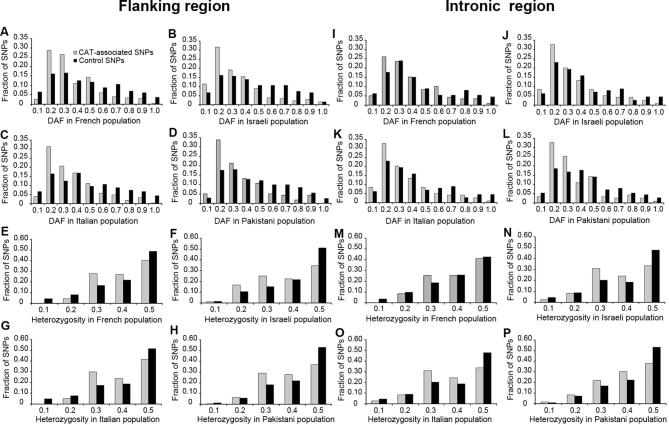
**DAF spectrums of CAT-associated SNPs in French, Israeli, Italian and Pakistani populations** DAF distributions of flanking region SNPs in French **(A)**, Israeli **(B)**, Italian **(C)**, and Pakistani populations **(D)**. Heterozygosity plots of flanking region SNPs in French **(E)**, Israeli **(F)**, Italian **(G)**, and Pakistani populations **(H)**. DAF distributions of intronic region SNPs in French **(I)**, Israeli **(J)**, Italian **(K)**, and Pakistani populations **(L)**. Heterozygosity plots of intronic region SNPs in French **(M)**, Israeli **(N)**, Italian **(O)**, and Pakistani populations **(P)**. DAF, derived allele frequency.

For the 12 HGDP-CEPH constituent populations, those residing in China, Japan, Russia, and Siberia were also suggested to have experienced balancing selection, whereas all four African populations could have experienced positive or purifying selection events (Figures S5 and S6). For the remaining populations, the French and Italian populations each showed a non-significant trend of balancing selection, whereas the populations residing in Israel and Pakistan remained ambiguous (Figure S7). Therefore, a clear world-wide selection pattern was revealed, indicating geographically specific genetic adaptation to CAT.

Next, *F*_ST_ was examined to measure the degree of population differentiation and to identify local adaptation signals. Among the four HGDP-CEPH continental populations, according to *F*_ST_ results derived from control SNPs, the population differentiation pattern was generally consistent with their speculated split time [31], with the European and Middle Eastern populations having the lowest *F*_ST_ value. However, for CAT-associated SNPs, the pattern was distorted with Asian and European populations having the lowest differentiation (Table S12). This trend was further confirmed in the HapMap data (Table S13). Several CAT SNPs were found to display high population differentiation values, namely between Asians and Africans (0.346–0.231) or between Europeans and Africans (0.277–0.218) as listed in Table S14.

**Supplementary Figure S8:**
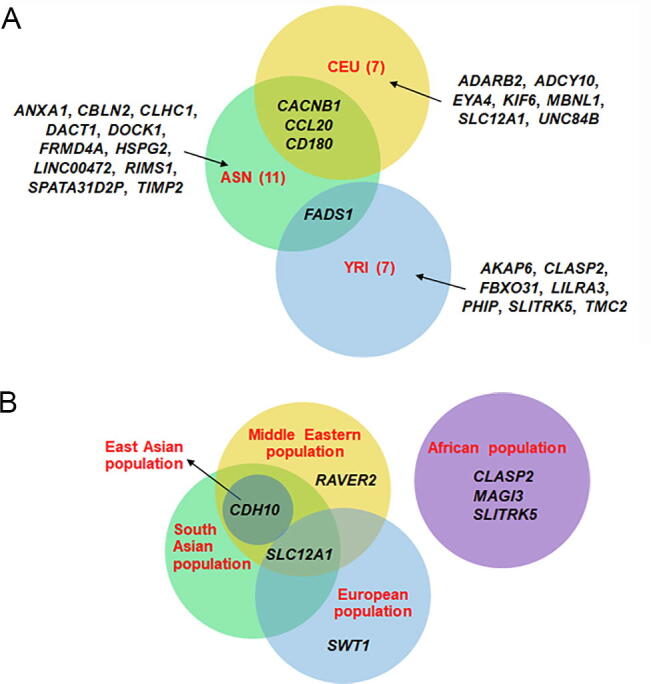
**Overlapping genes with high iHS scores among HapMap populations(A) and HGDP-CEPH populations (B)**

Finally, iHs were examined to identify recent and generally segregating selection sweeps. More SNPs were fixed in the African populations compared with controls, with no available iHs (*χ^2^*; *P* < 5 × 10^−5^). Additionally, distinct and shared positive selection signals were detected among different continental populations. For the HapMap populations, one SNP (rs174556 in *FADS1* gene) was shared between the ASN and YRI populations, and two SNPs (rs657672 in *CACNB1* and rs16823913 in *CCL20*) were shared between the ASN and CEU populations (Table S15). When only genes were considered, three genes (*CACNB1*, *CCL20*, and *CD180*) were shared in ASN and CEU populations, while only *FADS1* was shared between ASN and YRI populations (Figure S8A). In HGDP-CEPH populations, the signals were more focused on fewer genes, with one SNP (rs10942089 in *CDH10*) shared among the East Asian, Middle Eastern, and South Asian populations; another SNP (rs16960758 in *SLC12A1*) among the European, Middle Eastern, and South Asian populations; and a SNP (rs11208527 in *RAVER2*) between the European and Middle Eastern populations (Table S16). For selected genes, *CDH10* was shared among East/South Asian and Middle Eastern populations, while *SLC12A1* was shared between South Asian, Middle Eastern, and European populations. This left the African population with three distinct genes (*CLASP2*, *MAGI3*, and *SLITRK5*) (Figure S8B). Lastly, when the HapMap and HGDP-CEPH results were combined, only rs16960758 in *SLC12A1* for Europeans and rs439022 in *SLITRK5* for Africans, which are both eQTLs, were mutually verified in both datasets.

## Discussion

As the major source of the Earth's energy, the Sun has played a key role in the development as well as maintenance of a rich and diverse biosphere. Therefore, it has long been proposed as a strong driving force for the origin and subsequent evolution of human beings [32]. Morphological and physiological studies have revealed accumulating ethnic and clinal variations associated with each of the three main attributes of solar radiation: CAT, UVR, and SD [22], [33], [34]. However, no study thus far has examined these three factors simultaneously and the existing genetic studies can only explain a small fraction of the underlying heritability. This study sought to provide a full elucidation of the evolutionary role of solar radiation as a whole through a series of analyses with respect to CAT, UVR, and SD.

### CAT is the main driving force of solar radiation

Traditional selection analyses tended to identify ‘hard sweeps’ that *de novo* advantageous mutations with large effective size spread rapidly to fixation, usually agnostic to the underlying selective pressures. However, such ‘hard sweeps’ are rare. More adaptations may be expressed by the so-called ‘soft sweeps’ in which subtle allele frequency shifts occur in standing variation [35]. For this type, the environmental correlation approach provides a good alternative, further pinpointing the underlying driving force [36]. In this study, we combined the two types of methodologies together. First, the environmental correlation approach was used to select candidate loci for each attribute. Next, standard selection methods including *F*_ST_, iHS, and DAF were used to determine the selective scenario more specifically. By doing this, the signals and selection patterns revealed would be more accurate.

Furthermore, population structure is an important and unavoidable confounding factor. Generally, principal component (PC)-based analyses are used for correction, although the number of PCs included can be arbitrary and subjective. In this study, we carefully controlled for potential population structure in three ways. First, we included geographic coordinates, which have been confirmed as a good representative for human demographic history [37] as covariates in the multiple linear regression analysis for each SNP. Second, we corrected for null models derived from either genomic controls or deliberately defined signals. Finally, and most importantly, we verified our genome-wide signals experimentally using genetically more homogenous Chinese populations residing in multiple solar radiation environments and through confirmation in the dbCLINE database, which includes worldwide populations. By using these strategies, the identified selective signals are more likely to be truly related to UVR, CAT, and SD themselves.

Notably, CAT, especially an extremely minimum annual average temperature, is finally identified as the main force driving solar radiation selection of human genes involving development and immunity.

### Development and immunity genes are mostly selected by solar radiation

Previous studies have described a close association between human morphology and CAT, in accordance with Bergmann’s and Allen’s rules [9], [10]. In addition, annual average temperature has been found to be significantly correlated with body mass and relative sitting height [38]. These morphological variations are all related to efficient thermoregulation, from heat conservation to dissipation [33]. In this study, biological processes that occur at multiple levels, including cell differentiation, anatomical structure morphogenesis, organ development, and regulation of developmental processes, all showed significant enrichment in CAT-associated signals. This observation is supported by Frichot et al.’s work. They tested the same HGDP dataset with multiple temperature indexes through a latent factor mixed model and found a significant enrichment of GO terms associated with human developmental processes [39]. Next, genome-wide signals provide further information about the effect of CAT upon human development. Among the five genes derived from the six genome-wide CAT-associated signals, *ULBP3* and *KRT31* are involved in hair development [40], [41], while *DRD3* and *NTNG1* are involved in neuronal development [42], [43]. All six signals, except rs1107877 in *KRT31*, were successfully validated; five of them were significantly associated with the annual average CAT range and four were significantly associated with an annual average extremely minimum CAT. Previous studies on human skeletal variation have shown that the minimum temperature consistently had a stronger effect on shaping human morphology than maximum temperature [44], [45], and this was genetically supported by our results since most of our genome-wide CAT SNPs were more significantly associated with the annual average extremely minimum temperature or minimum temperature (winter). More interestingly, when the 316 CAT-associated genes were checked for GWAS traits, anthropometric traits, including height, weight (weight, obesity, adipose, and fat), waist-to-hip ratio, and BMI, demonstrated significant enrichment, especially in waist-hip ratio and obesity. Therefore, our overall results provide strong genomic evidence for Bergmann’s and Allen’s rules, further highlighting the role of low CAT upon human development.

Global variations in CAT have affected the distribution and abundance of disease vectors; accordingly, the resultant difference in infectious disease patterns could exert selective pressure on the human immune system [46]. This expectation has been partially verified as the pathogen selection hypothesis; that is, the local burden of infective agents would shape the variability of human genome, especially for immune-related genes [47]. The identified immunity clustering GO terms and enrichment of immunity-relevant GWAS-mapped traits within the CAT-associated SNPs provide genomic evidence linking CAT and immune system variations directly. This conclusion is also supported at the individual gene level. Because of pleiotropy, the protein encoded by *ULBP3* is also a ligand for the NKG2D receptor, and it can activate multiple signaling pathways in primary NK cells to produce cytokines and chemokines [48]. In addition, *DRD3-*encoded dopamine receptor D3 is important for modulating T cell function by inhibiting the activated T cell receptor-induced cell proliferation and secretion of IL-2, IFN-γ, and IL-4 [49]. Together, these results not only reveal a significant association between CAT and human immunity, but they also help elucidate CAT as the underlying environmental basis for the pathogen selection hypothesis.

In addition, UVR-associated signal *NADK* is involved in most reductive biosynthetic reactions and antioxidant defense systems by controlling the NADPH concentration [50], whereas *IGFBP3* is associated with osteoblast differentiation and bone development [51]. The UVR-associated signals can thus be genetically attributed to the developmental process and immunity as well.

### The underlying selection mechanism of solar radiation

As described in the recently modified Out-of-Africa dispersal model with minor introgression from archaic humans [52], human populations have lived in tropical Africa for the longest time; therefore, as they migrated and resided in temperate Eurasia and arctic Siberia, the declining CAT outside Africa would exert a new selective pressure [33]. Accordingly, our results suggest that the four African populations who lived within relatively invariant hot environments for the longest time (average CAT range from 27.0 °C–29.3 °C for the three tropical populations and 19.0 °C for South Africa) might have experienced ancient events of positive selection (complete sweeps), or they may have evolved under strict purifying selection. In addition, among the 401 CAT-associated SNPs, 49 were completely fixed, and more than half were nearly fixed in Africans. Taking into account these results along with the positively selected signals indicated by iHS and *F*_ST_ statistics for CAT SNPs, a selective scenario of positive selection upon several genes (by both hard sweep and soft sweep) together with events of purifying selection in other loci probably provide an optimal adaptation for African populations. As humans migrated out of Africa, the ancient heat-adapted genetic background encountered diverse CAT (average ranging from −10.0 °C in Siberia to 18.3 °C in Pakistan), particularly cold stress during the last glacial period [53]. Furthermore, the temperature fluctuates more fiercely throughout the year outside tropical Africa, usually with both hot summers and cold winters. Consistent with this change, five of the six CAT genome-wide signals were significantly associated with the annual average CAT range: four with annual average extremely minimum CAT and one with annual average maximum CAT. Therefore, under these conditions, balancing selection could probably result in more adaptation, even for the arctic Siberia population. Still, signals of positive selection, as indicated by iHS and *F*_ST_, may also have contributed to this local adaptation to CAT sweeping variants from low to intermediate frequencies. Here, positive selection would tend to act on pre-existing variations (selection on standing variation – soft-sweeps), except for the Siberian population. The situation, for Israeli and Pakistani populations, however, is ambiguous and may reflect complex interactions between natural selection and demographic history. Further studies with larger sample sizes and from more subpopulations are needed to better elucidate their selection patterns.

As for the associations between the morphological variations and CAT, all are related to efficient thermoregulation from heat conservation to dissipation, as described in Bergmann’s and Allen’s rules. Among the 316 CAT-associated genes, additional signals relevant to thermoregulation, including those involved in hair formation (*KRT31*, *KRT32*, *KRT35*, *KRTAP4-1*, *COL22A1*, *CSMD1*, and *OVOL1*), temperature homeostasis (*ACOT11* and *PPARGC1A*), and body fluid balance (*SLC12A1*), were highlighted. Here, solute carrier family 12 member 1 encoded by *SLC12A1* mediates Na^+^, K^+^, and Cl^−^ reabsorption. It also plays a vital role in the regulation of ionic balance and cell volume associated with sweating [54]. Recently, *SLC12A1* has been strongly suggested as a candidate gene for hypertension in a large-scale meta-analysis [55]. The derived rs16960758C allele in this gene may have undergone a strong positive selection among European, Middle Eastern, and South Asian populations, as indicated by iHS. Indeed, the loss of hair and a concomitant increase in the number of sweat glands are suggested to have been advantageous for sensitive whole-body cooling during early human evolution [56]. Collectively, these results provide evidence of genetic adaptation owing to thermoregulation [57].

Unlike previous evolutionary studies, which found a larger fraction of nonsynonymous SNPs [22], the identified CAT-associated signals in this study tended to be located more in flanking regions and introns. The GTEx project and HaploReg database were searched for the functional impact of the genome-wide SNPs. In total, five out of six CAT-associated signals and two of the UVR-associated signals all have important functional impact, either as *cis*- or *trans*-regulate genes or as modifiers of associated regulatory motifs. Therefore, the selection of CAT could be through gene regulation.

### Implication for future clinical studies

**Supplementary Figure S9:**
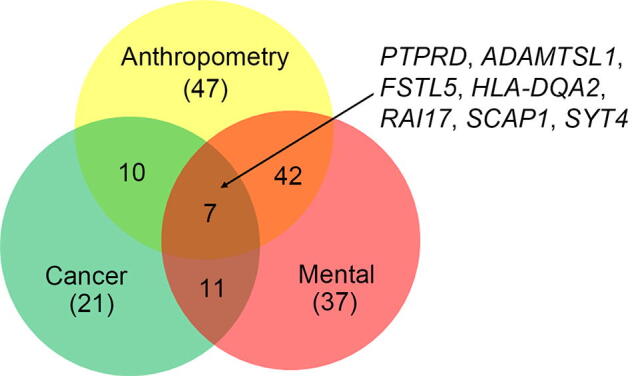
**Intersection results of the anthropometry, cancer,and mental related genes from CAT-associated genes**

For the CAT-associated signals, the following gene categories showed significant enrichment: immunity (92), hypertension and blood pressure (14), mental function and mental disorder (97), and cancer (49). This provides a new perspective for elucidating the genetic architecture and pathogenesis of these complex traits, as demonstrated by the examples of infectious diseases [58] and essential hypertension [59]. Generally, two main models were suggested to explain these observations. First, as proposed in the ancestral-susceptibility model, the prevalent alleles, most of which are ancestral and adaptive in Africa, may increase disease susceptibility, whereas the other alleles, which are mostly derived ones, are gradually becoming advantageous owing to the changing environment [60]. This model has been well explained in essential hypertension through the thrifty gene hypothesis [59], [61]. Second, genetic pleiotropy is responsible for both selected developmental processes and developmental disorders. This explanation is partially supported by the massive overlap between anthropometric genes and either mental or cancer related genes within the CAT signals (Figure S9). Meanwhile, selective disadvantages late in life accompanying a productive advantage at an early age will show themselves more clearly as human longevity increases [62]. Although the present results are insufficient to fully elucidate the evolutionary mechanisms for these complex traits, they offer an opportunity to connect associated genes and diseases with their underlying environmental factors, thereby providing new clinical perspectives.

## Conclusion

In summary, this is the first systematic study of the evolutionary effects of solar radiation on human genome diversity. CAT, especially cold stress, was identified as the main driving force of selection by solar radiation. Together with two other attributes, CAT mostly selected for genes involved in human development and immunity, with thermal regulation being the major mechanism. Specifically, the enrichment in developmental genes provides direct genomic evidence for Bergmann’s and Allen’s longstanding rules, and the enrichment in immunity categories reveals the underlying environmental driving force for the pathogen selection hypothesis. Furthermore, human populations residing in different environments and with different demographic histories showed region-specific selective patterns. This study thus provides an initial genetic insight into the evolutionary role of solar radiation in *Homo sapiens*; it also provides clues for future studies on humans and other species.

## Materials and methods

### Studied HGDP-CEPH human populations (*N* = 764)

Population genotype data were retrieved from the HGDP-CEPH dataset, which included 952 unrelated individuals from 52 populations [21]. Because more genetic data have accumulated for the HapMap YRI, CEU, CHB, and JPT populations that could be used for confirmation, all 40 HGDP-CEPH African, European, and Asian populations with specific climatic data were included (Biaka Pygmy, Cambodian, Mbuti Pygmy, Mozabite, and San were excluded owing to a lack of specific climatic data). Genetic data for these 40 populations, which were further merged into 12 integrated countries/regions (*N* = 764, Table S11) to enhance representativeness [15], were used for data analysis.

### Replicating Chinese human populations (*N* = 1395)

**Supplementary Figure S10:**
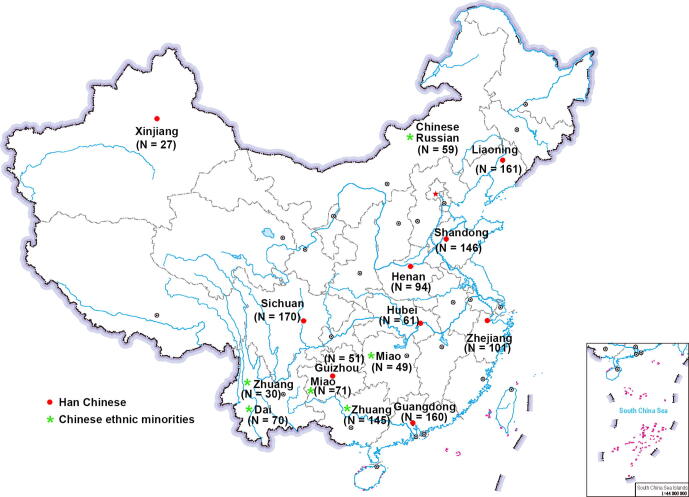
**Schematic representation of sampling for Chinese populations** The Han individuals were sampled randomly from nine provinces of China (*N* = 971, denoted by •), while the four Chinese ethnic minorities were from six resident areas (*N* = 424, denoted by *).

To better control for the underlying population structure, the results from the HGDP-CEPH populations were validated in a replication sample set of Chinese populations (Table S17). A total of 971 Han individuals were sampled randomly from nine provinces of China, and the four Chinese ethnic minorities were from six resident areas (Figure S10, *N* = 424). The geographic origin of each individual was ascertained by self-reported precoded geographic tenancy for three generations, and only individuals without a history of family migration were used.

### Climatological and geographic information

For HGDP-CEPH populations (Table S11), environmental data, including the daily average SD, CAT, and altitude, were extracted from the World Meteorological Organization [15] according to the sampling latitude and longitude data provided [21]. UVR (UV minimum erythema dose, UVMED) data were derived from readings taken from the NASA Total Ozone Mapping Spectrometer (TOMS) [3]. For replicating Chinese populations, climatological data (daily average SD; annual average CAT, maximum/minimum CAT, CAT range, extreme maximum/minimum CAT; annual and monthly average UVR dose) were collected from the China Meteorological Data Sharing Service System according to geographic information (altitude, latitude, and longitude) [15]. For all populations, if specific climatic information was not available, information for the closest neighboring site was used as a substitute. In addition, an average would be taken if samples were collected from more than one nearby location. All the climatic data were demonstrated in Tables S18 and S19, and all geographic data were transformed into natural logarithm form to ensure normality.

### SNP genotyping for replication

Six genome-wide CAT-associated SNPs (rs1107877, rs11185115, rs12202737, rs12626864, rs13729, and rs9825563) were experimentally confirmed in Chinese populations. Genomic DNA was extracted from whole blood using the standard phenol/chloroform method. Among them, rs11185115 within *NTNG1* was replaced by a nearby SNP, rs1444041 (*r^2^* = 0.954), which was not in the original dataset, owing to a lack of the appropriate SNPstream primers; rs12202737 was represented by rs13729 from the same *ULBP3* gene because of their complete LD in the CHB population (*r^2^* = 1). In total, four SNPs (rs12626864, rs13729, rs1444041, and rs9825563) were genotyped using the Beckman SNPstream genotyping system [63], while one SNP (rs1107877) was genotyped by the PCR-RFLP method (Sau3AI, Takara, China). For 2 UVR-associated SNPs, rs1586360 was not genotyped owing to its low MAF in the CHB populations (MAF < 0.1). Only rs7531583 was genotyped using the same Beckman SNPstream genotyping system.

### GWAdS data analyses

**Supplementary Figure S11:**
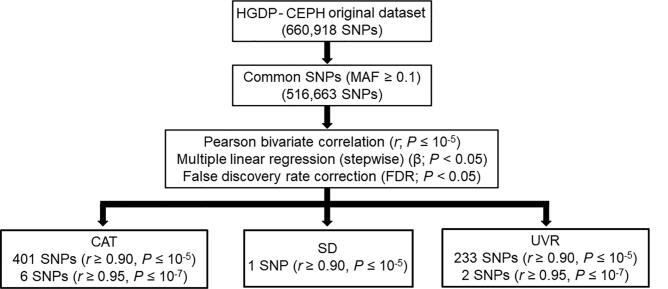
**Flowchart of the GWAdS against CAT, UVR, and SD using the HGDP-CEPH data** First, 516,663 common SNPs (MAF ≥ 0.1) were chosen from the whole 660,918 SNPs in the original HGDP-CEPH dataset. Secondly, for each common SNP, Pearson bivariate correlation and multiple linear regression analysis were used to evaluate the association between allele frequencies and each of the three solar radiation attributes among the 12 HGDP-CEPH countries/regions. Next, FDR was explored to correct for whole genome testing. After that, associated SNPs for each solar radiation attributes were obtained. GWAdS, genome-wide adaptation studies.

For each of the 516,663 common SNPs (MAF ≥ 0.1) of the whole 660,918 SNPs in the original HGDP-CEPH dataset [21], allelic frequencies were calculated for the 12 HGDP-CEPH countries/regions. Pearson bivariate correlation was then carried out to evaluate the association between allele frequencies and each of the three solar radiation attributes. For each solar radiation attribute, any significant Pearson bivariate correlation was further confirmed by multiple linear regression analysis using the three geographic parameters and the other two attributes as covariates (SPSS for Windows; 16.0). A modified positive false discovery rate, pFDR, was explored to correct for whole genome testing [64], and the WGAViewer software [65] was used to draw Manhattan plots to show the locations of the significant signals (see the flowchart, Figure S11).

### GWAS mapping and functional clustering

Associated genes were assigned according to the dbSNP database, and SNPs within an intergenic region were annotated with the closest gene symbol. Then, they were mapped to GO annotation terms [23] and KEGG pathways [24] using the publicly available program Database for Annotation, Visualization, and Integrated Discovery (DAVID) [66]. Here, the GO and KEGG results of all 17,169 HGDP-CEPH dataset genes were used as null distributions. In addition, these associated genes were checked in the NHGRI-EBI GWAS Catalog (http://www.ebi.ac.uk/gwas/search) through 20 May 2016 for potential phenotype effect. Similarly, the GWAS category distribution patterns for both the 12,456 total GWAS mapped genes and the 17,169 HGDP-CEPH genes were used as two separate null distributions.

### Population genetics analysis

In addition to the HGDP-CEPH data, allele frequencies for the HapMap CHB, JPT, CEU, and YRI populations retrieved from HapMart (HapMap_rel27 dat) were analyzed. First, in DAF analysis, SNPs for CAT-associated intronic and flanking region SNPs were performed separately, with specific controls defined from the same Illumina 650Y dataset. For flanking region SNPs, controls should be from unassociated genes; the physical distance should be similar to that of the associated SNP, with matches made in a one-to-one manner. For intronic SNPs, we first carried out a similar one-to-one matching. Then we performed 100 simulations by randomly re-sampling the intronic SNPs from the remaining unassociated genes, regardless of physical distance. For all these SNPs, the ancestral allele information was retrieved from dbSNP. Second, pairwise *F*_ST_ values [29] for each SNP were calculated to evaluate the degree of population differentiation and identify local adaptation signals. Third, for each SNP, the iHS was retrieved from Haplotter [30] for HapMap data and from the HGDP Selection Browser [67] for HGDP-CEPH data, with an |iHS| ≥ 2.0 used to infer positive selection signals. Notably, the control SNPs defined in the DAF analysis were also used as controls in the *F*_ST_ and iHS analyses.

### Statistical analysis for Chinese replication populations

HWE was tested through the software PEDSTATS [68]. Statistical analysis of the gender and age differences of allele frequencies was performed by *χ^2^* tests (SPSS for Windows; 16.0). Environmental associations were assessed using Pearson bivariate correlation and multiple linear regression analysis as described for the worldwide populations (SPSS for Windows; 16.0).

## Ethical statement

Blood samples from all 1395 Chinese individuals for the confirmation experiment were obtained with written informed consent. The protocol for this study was reviewed and approved by the ethics committee of the Kunming Institute of Zoology, Chinese Academy of Sciences.

## CRediT author statement

**Lindan Ji:** Investigation, Formal analysis, Writing - original draft, Writing - review & editing. **Dongdong Wu:** Formal analysis, Writing - original draft. **Haibing Xie:** Formal analysis, Writing - original draft. **Binbin Yao:** Formal analysis. **Yanming Chen:** Formal analysis. **David M. Irwin:** Writing - original draft, Writing - review & editing. **Dan Huang:** Formal analysis. **Jin Xu:**  Conceptualization, Supervision, Formal analysis, Writing - original draft, Writing - review & editing. **Nelson L.S. Tang:** Conceptualization, Supervision, Formal analysis, Writing - original draft, Writing - review & editing. **Yaping Zhang**:  Conceptualization, Supervision, Writing - original draft, Writing - review & editing. All authors read and approved the final manuscript.

## Competing interests

The authors have declared that no competing interests exist.
