# Supplementary material for: Ambient Temperature is A Strong Selective Factor Influencing Human Development and Immunity
Source: Genomics Proteomics Bioinformatics. 2020 Aug 19;18(5):489–500. doi: 10.1016/j.gpb.2019.11.009 (PMC8377383; doi:10.1016/j.gpb.2019.11.009)
Supplement: Supplementary Table S1 [file mmc1.doc]

**Table S1 CAT-associated SNPs at the suggestive 1×10^-5^ level**

| **Name** | **Rank** | **Chr** | **Gene symbol** | **Location** | ***r*** | ***P*** |
| --- | --- | --- | --- | --- | --- | --- |
| rs12202737 | 1 | 6 | *ULBP3* | intron | 0.9846 | 6.73×10^-9^ |
| rs13729 | 2 | 6 | *ULBP3* | flanking_3UTR | 0.9839 | 8.37×10^-9^ |
| rs1107877 | 3 | 17 | *KRT31* | flanking_3UTR | 0.9833 | 1.01×10^-8^ |
| rs12626864 | 4 | 21 | *LINC00112* | flanking_5UTR | 0.9815 | 1.66×10^-8^ |
| rs9825563 | 5 | 3 | *DRD3* | flanking_5UTR | 0.9766 | 5.26×10^-8^ |
| rs11185115 | 6 | 1 | *NTNG1* | intron | 0.9758 | 6.26×10^-8^ |
| rs10764 | 7 | 3 | *CLASP2* | 3UTR | 0.9730 | 1.08×10^-7^ |
| rs2051579 | 8 | 22 | *RBFOX2* | intron | 0.9723 | 1.22×10^-7^ |
| rs12215953 | 9 | 6 | *IRAK1BP1* | flanking_5UTR | 0.9703 | 1.73×10^-7^ |
| rs4804662 | 10 | 19 | *C19orf45* | flanking_3UTR | 0.9700 | 1.81×10^-7^ |
| rs6987910 | 11 | 8 | *MSRA* | flanking_5UTR | 0.9685 | 2.31×10^-7^ |
| rs17059327 | 12 | 9 | *ANXA1* | flanking_3UTR | 0.9672 | 2.83×10^-7^ |
| rs1016145 | 13 | 12 | *PDE3A* | intron | 0.9668 | 3.02×10^-7^ |
| rs12600182 | 14 | 16 | *FOXF1* | flanking_5UTR | 0.9659 | 3.43×10^-7^ |
| rs7048129 | 15 | 9 | *KIAA1958* | flanking_5UTR | 0.9655 | 3.62×10^-7^ |
| rs10188319 | 16 | 2 | *MYADML* | flanking_5UTR | 0.9654 | 3.71×10^-7^ |
| rs12483420 | 17 | 21 | *BACE2* | flanking_5UTR | 0.9652 | 3.77×10^-7^ |
| rs2185217 | 18 | 1 | *PBX1* | intron | 0.9648 | 3.99×10^-7^ |
| rs12591340 | 19 | 15 | *ARRDC4* | flanking_5UTR | 0.9637 | 4.69×10^-7^ |
| rs1003406 | 20 | 8 | *JPH1* | intron | 0.9633 | 4.94×10^-7^ |
| rs1003260 | 21 | 6 | *RIMS1* | flanking_5UTR | 0.9616 | 6.14×10^-7^ |
| rs9303058 | 22 | 17 | *MYOCD* | intron | 0.9603 | 7.30×10^-7^ |
| rs4235269 | 23 | 4 | *LOC389199* | flanking_3UTR | 0.9602 | 7.36×10^-7^ |
| rs16959558 | 24 | 16 | *BBS2* | flanking_5UTR | 0.9600 | 7.52×10^-7^ |
| rs11733383 | 25 | 4 | *C4orf50* | intron | 0.9600 | 7.53×10^-7^ |
| rs2684000 | 26 | 8 | *MSRA* | flanking_5UTR | 0.9599 | 7.67×10^-7^ |
| rs17283269 | 27 | 3 | *SUCNR1* | flanking_3UTR | 0.9598 | 7.70×10^-7^ |
| rs7647008 | 28 | 3 | *CLASP2* | intron | 0.9597 | 7.78×10^-7^ |
| rs7502935 | 29 | 17 | *TIMP2* | intron | 0.9592 | 8.29×10^-7^ |
| rs11707430 | 30 | 3 | *LPP* | intron | 0.9590 | 8.50×10^-7^ |
| rs758522 | 31 | 17 | *FLJ42842* | flanking_3UTR | 0.9586 | 8.89×10^-7^ |
| rs12469080 | 32 | 2 | *YPEL5* | flanking_3UTR | 0.9585 | 9.05×10^-7^ |
| rs9646637 | 33 | 19 | *UQCRFS1* | flanking_3UTR | 0.9582 | 9.40×10^-7^ |
| rs2707772 | 34 | 5 | *CD180* | flanking_5UTR | 0.9574 | 1.03×10^-6^ |
| rs11780158 | 35 | 8 | *CCDC25* | flanking_3UTR | 0.9570 | 1.07×10^-6^ |
| rs16842480 | 36 | 4 | *FLJ45966* | flanking_3UTR | 0.9568 | 1.11×10^-6^ |
| rs2420309 | 37 | 10 | *VAX1* | flanking_5UTR | 0.9564 | 1.16×10^-6^ |
| rs1200610 | 38 | 1 | *SWT1* | intron | 0.9563 | 1.17×10^-6^ |
| rs1122073 | 39 | 17 | *PMP22* | flanking_3UTR | 0.9563 | 1.17×10^-6^ |
| rs4074143 | 40 | 4 | *SLIT2* | flanking_5UTR | 0.9563 | 1.17×10^-6^ |
| rs9664222 | 41 | 10 | *MINPP1* | flanking_3UTR | 0.9562 | 1.19×10^-6^ |
| rs2406669 | 42 | X | *DMD* | flanking_5UTR | 0.9560 | 1.21×10^-6^ |
| rs16833435 | 43 | 3 | *ATP11B* | flanking_5UTR | 0.9556 | 1.26×10^-6^ |
| rs4540044 | 44 | 4 | *SLIT2* | flanking_5UTR | 0.9555 | 1.27×10^-6^ |
| rs9402494 | 45 | 6 | *EYA4* | intron | 0.9551 | 1.33×10^-6^ |
| rs473170 | 46 | 3 | *MBNL1* | flanking_5UTR | 0.9548 | 1.38×10^-6^ |
| rs10953303 | 47 | 7 | *ZAN* | coding | 0.9547 | 1.39×10^-6^ |
| rs556925 | 48 | 3 | *MBNL1* | flanking_5UTR | 0.9547 | 1.39×10^-6^ |
| rs2726554 | 49 | 8 | *TOX* | intron | 0.9547 | 1.40×10^-6^ |
| rs17139596 | 50 | 16 | *RBFOX1* | intron | 0.9546 | 1.41×10^-6^ |
| rs1354033 | 51 | 13 | *SLITRK6* | flanking_3UTR | 0.9543 | 1.45×10^-6^ |
| rs6545433 | 52 | 2 | *SPTBN1* | intron | 0.9543 | 1.45×10^-6^ |
| rs11208527 | 53 | 1 | *RAVER2* | intron | 0.9542 | 1.47×10^-6^ |
| rs7806458 | 54 | 7 | *TMEM176B* | flanking_3UTR | 0.9542 | 1.47×10^-6^ |
| rs4859571 | 55 | 4 | *ASAHL* | coding | 0.9542 | 1.47×10^-6^ |
| rs2933352 | 56 | 12 | *CNTN1* | flanking_5UTR | 0.9541 | 1.49×10^-6^ |
| rs16960758 | 57 | 15 | *SLC12A1* | flanking_3UTR | 0.9541 | 1.49×10^-6^ |
| rs11047130 | 58 | 12 | *SOX5* | intron | 0.9540 | 1.5E-06 |
| rs2826486 | 59 | 21 | *NCAM2* | flanking_5UTR | 0.9539 | 1.52×10^-6^ |
| rs3843979 | 60 | 3 | *PLD1* | flanking_5UTR | 0.9538 | 1.53×10^-6^ |
| rs11603089 | 61 | 11 | *SAA1* | flanking_5UTR | 0.9535 | 1.58×10^-6^ |
| rs8110572 | 62 | 19 | *ATCAY* | flanking_5UTR | 0.9532 | 1.64×10^-6^ |
| rs241981 | 63 | 12 | *EFCAB4B* | intron | 0.9530 | 1.66×10^-6^ |
| rs4660632 | 64 | 1 | *FAM80A* | flanking_3UTR | 0.9530 | 1.67×10^-6^ |
| rs4768290 | 65 | 12 | *CNTN1* | flanking_5UTR | 0.9529 | 1.69E-06 |
| rs6927143 | 66 | 6 | *EYA4* | flanking_5UTR | 0.9528 | 1.70×10^-6^ |
| rs9571952 | 67 | 13 | *KLHL1* | flanking_3UTR | 0.9528 | 1.70×10^-6^ |
| rs3983739 | 68 | 12 | *GSG1* | flanking_5UTR | 0.9528 | 1.70×10^-6^ |
| rs2829679 | 69 | 21 | *LINC00158* | flanking_3UTR | 0.9527 | 1.72×10^-6^ |
| rs156769 | 70 | 9 | *ANXA1* | flanking_3UTR | 0.9527 | 1.72×10^-6^ |
| rs1531160 | 71 | 3 | *UBE2E2* | flanking_5UTR | 0.9526 | 1.74×10^-6^ |
| rs767739 | 72 | 20 | *TMC2* | intron | 0.9525 | 1.76×10^-6^ |
| rs11059603 | 73 | 12 | *SLC15A4* | flanking_3UTR | 0.9524 | 1.77×10^-6^ |
| rs2825324 | 74 | 21 | *PRSS7* | flanking_5UTR | 0.9524 | 1.77×10^-6^ |
| rs11655712 | 75 | 17 | *KRT32* | flanking_5UTR | 0.9523 | 1.79×10^-6^ |
| rs17079315 | 76 | 8 | *CSMD1* | intron | 0.9522 | 1.82×10^-6^ |
| rs4796143 | 77 | 17 | *CCL16* | flanking_3UTR | 0.9521 | 1.82×10^-6^ |
| rs10932022 | 78 | 2 | *CD28* | flanking_3UTR | 0.9521 | 1.83×10^-6^ |
| rs1342517 | 79 | 15 | *MCTP2* | flanking_5UTR | 0.9521 | 1.84E-06 |
| rs883607 | 80 | 4 | *FLJ45966* | flanking_3UTR | 0.9520 | 1.86×10^-6^ |
| rs10444381 | 81 | 11 | *RAB30* | flanking_5UTR | 0.9519 | 1.87×10^-6^ |
| rs17083869 | 82 | 13 | *KLHL1* | flanking_3UTR | 0.9518 | 1.89×10^-6^ |
| rs325781 | 83 | 3 | *MBNL1* | flanking_5UTR | 0.9518 | 1.90×10^-6^ |
| rs9571953 | 84 | 13 | *KLHL1* | flanking_3UTR | 0.9518 | 1.90×10^-6^ |
| rs961328 | 85 | 2 | *ETAA1* | flanking_5UTR | 0.9517 | 1.92×10^-6^ |
| rs6768189 | 86 | 3 | *CACNA2D3* | intron | 0.9513 | 1.98×10^-6^ |
| rs7920517 | 87 | 10 | *MSMB* | flanking_5UTR | 0.9513 | 1.99×10^-6^ |
| rs2049791 | 88 | 14 | *TRAV9-2* | flanking_3UTR | 0.9512 | 2.00×10^-6^ |
| rs11119208 | 89 | 1 | *CAMK1G* | flanking_5UTR | 0.9512 | 2.01×10^-6^ |
| rs244132 | 90 | 21 | *LINC00308* | flanking_3UTR | 0.9511 | 2.02×10^-6^ |
| rs1990367 | 91 | 7 | *GLCCI1* | flanking_5UTR | 0.9511 | 2.03×10^-6^ |
| rs2613577 | 92 | 7 | *DOCK4* | flanking_3UTR | 0.9511 | 2.04×10^-6^ |
| rs10889675 | 93 | 1 | *IL23R* | intron | 0.9510 | 2.04×10^-6^ |
| rs12449970 | 94 | 17 | *SLC39A11* | intron | 0.9509 | 2.07×10^-6^ |
| rs731900 | 95 | 8 | *POU5F1B* | flanking_5UTR | 0.9506 | 2.13×10^-6^ |
| rs1959049 | 96 | 14 | *BDKRB1* | flanking_3UTR | 0.9506 | 2.13×10^-6^ |
| rs17197662 | 97 | 14 | *TRAV8-6* | flanking_5UTR | 0.9504 | 2.18×10^-6^ |
| rs2222924 | 98 | 14 | *TRAV12-3* | flanking_3UTR | 0.9504 | 2.18×10^-6^ |
| rs2014007 | 99 | 2 | *VRK2* | flanking_5UTR | 0.9504 | 2.18×10^-6^ |
| rs6049825 | 100 | 20 | *SYNDIG1* | intron | 0.9503 | 2.19×10^-6^ |
| rs12982016 | 101 | 19 | *NLRP13* | intron | 0.9503 | 2.21×10^-6^ |
| rs10279772 | 102 | 7 | *AMPH* | flanking_5UTR | 0.9502 | 2.23×10^-6^ |
| rs4952098 | 103 | 2 | *YPEL5* | flanking_5UTR | 0.9500 | 2.26×10^-6^ |
| rs12426878 | 104 | 12 | *PTPRO* | intron | 0.9500 | 2.27×10^-6^ |
| rs2237428 | 105 | 7 | *GLI3* | intron | 0.9498 | 2.30×10^-6^ |
| rs6860994 | 106 | 5 | *CDH10* | flanking_5UTR | 0.9497 | 2.32×10^-6^ |
| rs17580847 | 107 | 17 | *KRT35* | flanking_5UTR | 0.9497 | 2.34×10^-6^ |
| rs10895522 | 108 | 11 | *PDGFD* | flanking_3UTR | 0.9496 | 2.35×10^-6^ |
| rs16909659 | 109 | 11 | *FANCF* | flanking_3UTR | 0.9496 | 2.36×10^-6^ |
| rs734512 | 110 | 10 | *ZNF248* | flanking_3UTR | 0.9496 | 2.36×10^-6^ |
| rs995491 | 111 | 5 | *CDH18* | intron | 0.9495 | 2.37×10^-6^ |
| rs82 | 112 | 7 | *PHF14* | flanking_3UTR | 0.9495 | 2.37×10^-6^ |
| rs966498 | 113 | 2 | *YPEL5* | flanking_3UTR | 0.9494 | 2.39×10^-6^ |
| rs2189947 | 114 | 7 | *NDUFA4* | flanking_3UTR | 0.9493 | 2.43×10^-6^ |
| rs2375278 | 115 | 1 | *SYF2* | flanking_3UTR | 0.9492 | 2.44×10^-6^ |
| rs2704517 | 116 | 10 | *COL13A1* | flanking_5UTR | 0.9491 | 2.47×10^-6^ |
| rs4658931 | 117 | 1 | *FAM89A* | flanking_5UTR | 0.9490 | 2.50×10^-6^ |
| rs10783046 | 118 | 1 | *PTBP2* | flanking_5UTR | 0.9489 | 2.51×10^-6^ |
| rs956187 | 119 | 1 | *PTBP2* | flanking_5UTR | 0.9489 | 2.51×10^-6^ |
| rs17233751 | 120 | 2 | *SP3* | flanking_3UTR | 0.9486 | 2.60×10^-6^ |
| rs7333303 | 121 | 13 | *SLITRK6* | flanking_5UTR | 0.9482 | 2.70×10^-6^ |
| rs4763422 | 122 | 12 | *RERG* | flanking_5UTR | 0.9481 | 2.71×10^-6^ |
| rs10488782 | 123 | 11 | *DSCAML1* | flanking_5UTR | 0.9481 | 2.73×10^-6^ |
| rs2968800 | 124 | 2 | *CLHC1* | flanking_3UTR | 0.9480 | 2.74×10^-6^ |
| rs17523194 | 125 | 5 | *CDH10* | flanking_5UTR | 0.9480 | 2.75×10^-6^ |
| rs4403628 | 126 | 1 | *DUSP23* | flanking_3UTR | 0.9480 | 2.75×10^-6^ |
| rs1999290 | 127 | 21 | *NCAM2* | flanking_5UTR | 0.9479 | 2.78×10^-6^ |
| rs17023344 | 128 | 1 | *WARS2* | flanking_5UTR | 0.9478 | 2.80×10^-6^ |
| rs6438101 | 129 | 3 | *CD200R1L* | flanking_3UTR | 0.9476 | 2.85×10^-6^ |
| rs696856 | 130 | 3 | *DHX36* | flanking_5UTR | 0.9473 | 2.92×10^-6^ |
| rs3767141 | 131 | 1 | *HSPG2* | intron | 0.9472 | 2.97×10^-6^ |
| rs12441320 | 132 | 15 | *ATP10A* | flanking_5UTR | 0.9468 | 3.06×10^-6^ |
| rs138705 | 133 | 22 | *UNC84B* | intron | 0.9466 | 3.11×10^-6^ |
| rs12231481 | 134 | 12 | *SOX5* | intron | 0.9466 | 3.12×10^-6^ |
| rs876121 | 135 | 6 | *CYP39A1* | flanking_3UTR | 0.9465 | 3.17×10^-6^ |
| rs9289423 | 136 | 3 | *ACPP* | flanking_5UTR | 0.9464 | 3.17×10^-6^ |
| rs9842169 | 137 | 3 | *ROBO2* | flanking_3UTR | 0.9464 | 3.19×10^-6^ |
| rs1361812 | 138 | 1 | *EDG1* | flanking_3UTR | 0.9463 | 3.21×10^-6^ |
| rs2278959 | 139 | 3 | *OSBPL10* | coding | 0.9462 | 3.23×10^-6^ |
| rs2915930 | 140 | 19 | *ZNF676* | flanking_5UTR | 0.9462 | 3.24×10^-6^ |
| rs1538977 | 141 | 1 | *DISC1* | intron | 0.9462 | 3.25×10^-6^ |
| rs17049031 | 142 | 4 | *PCDH18* | flanking_3UTR | 0.9461 | 3.27×10^-6^ |
| rs7256835 | 143 | 19 | *ATCAY* | flanking_5UTR | 0.9461 | 3.28×10^-6^ |
| rs1350380 | 144 | 16 | *GPR139* | flanking_3UTR | 0.9460 | 3.29×10^-6^ |
| rs2098469 | 145 | 12 | *GRIN2B* | intron | 0.9459 | 3.33×10^-6^ |
| rs1891086 | 146 | 6 | *PRL* | flanking_3UTR | 0.9459 | 3.34×10^-6^ |
| rs717984 | 147 | 14 | *ACIN1* | intron | 0.9458 | 3.35×10^-6^ |
| rs1727668 | 148 | 6 | *LINC00472* | intron | 0.9458 | 3.36×10^-6^ |
| rs7667982 | 149 | 4 | *LOC389199* | flanking_5UTR | 0.9458 | 3.37×10^-6^ |
| rs1520 | 150 | 6 | *KIF6* | intron | 0.9457 | 3.38×10^-6^ |
| rs17231602 | 151 | 10 | *ATOH7* | flanking_5UTR | 0.9457 | 3.41×10^-6^ |
| rs10895517 | 152 | 11 | *PDGFD* | flanking_3UTR | 0.9454 | 3.47×10^-6^ |
| rs1976393 | 153 | 3 | *OPA1* | flanking_3UTR | 0.9454 | 3.49×10^-6^ |
| rs7214006 | 154 | 17 | *KRTAP4-1* | flanking_5UTR | 0.9454 | 3.49×10^-6^ |
| rs951780 | 155 | 8 | *TNKS* | flanking_3UTR | 0.9453 | 3.51×10^-6^ |
| rs12970508 | 156 | 18 | *LDLRAD4* | flanking_5UTR | 0.9453 | 3.51×10^-6^ |
| rs11608383 | 157 | 12 | *NEDD1* | flanking_3UTR | 0.9451 | 3.57×10^-6^ |
| rs394028 | 158 | 13 | *SLITRK5* | flanking_3UTR | 0.9451 | 3.58×10^-6^ |
| rs722490 | 159 | 11 | *FANCF* | flanking_3UTR | 0.9451 | 3.59×10^-6^ |
| rs1931708 | 160 | 10 | *DOCK1* | flanking_3UTR | 0.9450 | 3.62×10^-6^ |
| rs11026668 | 161 | 11 | *FANCF* | flanking_3UTR | 0.9449 | 3.66×10^-6^ |
| rs2325890 | 162 | 20 | *TMC2* | intron | 0.9449 | 3.66×10^-6^ |
| rs7944110 | 163 | 11 | *RREB1* | flanking_3UTR | 0.9448 | 3.67×10^-6^ |
| rs4910295 | 164 | 11 | *GALNTL4* | intron | 0.9445 | 3.79×10^-6^ |
| rs977799 | 165 | 1 | *NTNG1* | intron | 0.9444 | 3.82×10^-6^ |
| rs3929518 | 166 | 3 | *PLD1* | flanking_5UTR | 0.9443 | 3.84×10^-6^ |
| rs317373 | 167 | 17 | *CCL2* | flanking_5UTR | 0.9442 | 3.88×10^-6^ |
| rs1339959 | 168 | 22 | *TMEM191A* | flanking_5UTR | 0.9441 | 3.92×10^-6^ |
| rs762308 | 169 | 3 | *ITGA9* | intron | 0.9440 | 3.94×10^-6^ |
| rs11227306 | 170 | 11 | *OVOL1* | flanking_3UTR | 0.9440 | 3.96×10^-6^ |
| rs2528679 | 171 | 7 | *DOCK4* | flanking_3UTR | 0.9439 | 3.98×10^-6^ |
| rs12117596 | 172 | 1 | *SLC35F3* | intron | 0.9438 | 4.02×10^-6^ |
| rs5932550 | 173 | X | *SMARCA1* | flanking_3UTR | 0.9438 | 4.03×10^-6^ |
| rs7328476 | 174 | 13 | *SLITRK5* | flanking_3UTR | 0.9437 | 4.06×10^-6^ |
| rs6453492 | 175 | 5 | *MTX3* | flanking_3UTR | 0.9435 | 4.12×10^-6^ |
| rs1979469 | 176 | 4 | *ELOVL6* | intron | 0.9435 | 4.13×10^-6^ |
| rs13356966 | 177 | 5 | *CDH18* | flanking_5UTR | 0.9434 | 4.17×10^-6^ |
| rs1994766 | 178 | 16 | *A2BP1* | intron | 0.9434 | 4.18×10^-6^ |
| rs2981579 | 179 | 10 | *FGFR2* | intron | 0.9433 | 4.18×10^-6^ |
| rs10903595 | 180 | 10 | *ADARB2* | flanking_5UTR | 0.9433 | 4.18×10^-6^ |
| rs4999155 | 181 | 9 | *SPATA31D2P* | flanking_5UTR | 0.9433 | 4.18×10^-6^ |
| rs16975420 | 182 | 13 | *COL4A1* | intron | 0.9433 | 4.21×10^-6^ |
| rs3772583 | 183 | 3 | *GYG1* | intron | 0.9433 | 4.21×10^-6^ |
| rs4370521 | 184 | 8 | *C8orf4* | flanking_5UTR | 0.9433 | 4.21×10^-6^ |
| rs1713736 | 185 | 8 | *TBC1D31* | flanking_3UTR | 0.9431 | 4.26×10^-6^ |
| rs4556079 | 186 | 8 | *COL22A1* | flanking_5UTR | 0.9430 | 4.32×10^-6^ |
| rs591269 | 187 | 6 | *CITED2* | flanking_5UTR | 0.9429 | 4.33×10^-6^ |
| rs10511138 | 188 | 3 | *C3orf38* | flanking_3UTR | 0.9429 | 4.34×10^-6^ |
| rs10943606 | 189 | 6 | *PHIP* | intron | 0.9428 | 4.39×10^-6^ |
| rs13039817 | 190 | 20 | *NPBWR2* | flanking_5UTR | 0.9428 | 4.39×10^-6^ |
| rs495296 | 191 | 1 | *KCNK2* | flanking_5UTR | 0.9428 | 4.39×10^-6^ |
| rs2471552 | 192 | 7 | *IGFBP3* | flanking_5UTR | 0.9427 | 4.40×10^-6^ |
| rs32935 | 193 | 5 | *ARAP3* | flanking_5UTR | 0.9426 | 4.44×10^-6^ |
| rs2825319 | 194 | 21 | *PRSS7* | flanking_5UTR | 0.9426 | 4.45×10^-6^ |
| rs12097635 | 195 | 1 | *FAM78B* | intron | 0.9426 | 4.47×10^-6^ |
| rs2025615 | 196 | 1 | *KLF17* | flanking_5UTR | 0.9425 | 4.50×10^-6^ |
| rs7522642 | 197 | 1 | *KLF17* | flanking_5UTR | 0.9425 | 4.50×10^-6^ |
| rs598654 | 198 | 2 | *TMEM163* | intron | 0.9422 | 4.60×10^-6^ |
| rs1025806 | 199 | 1 | *EFCAB14* | flanking_3UTR | 0.9421 | 4.64×10^-6^ |
| rs32018 | 200 | 5 | *CD180* | flanking_5UTR | 0.9421 | 4.64×10^-6^ |
| rs17034109 | 201 | 3 | *CNTN6* | intron | 0.9420 | 4.67×10^-6^ |
| rs4909062 | 202 | 7 | *PTPRN2* | intron | 0.9420 | 4.68×10^-6^ |
| rs1554481 | 203 | 2 | *OTOF* | intron | 0.9420 | 4.70×10^-6^ |
| rs12889403 | 204 | 14 | *APOPT1* | intron | 0.9420 | 4.71E-06 |
| rs11694535 | 205 | 2 | *ADAM23* | intron | 0.9419 | 4.71×10^-6^ |
| rs9907295 | 206 | 17 | *RDM1* | intron | 0.9418 | 4.77×10^-6^ |
| rs3753658 | 207 | 1 | *EPHX1* | flanking_5UTR | 0.9418 | 4.78×10^-6^ |
| rs10800356 | 208 | 1 | *TBX19* | flanking_3UTR | 0.9418 | 4.79×10^-6^ |
| rs2235588 | 209 | 20 | *ZMYND8* | intron | 0.9415 | 4.88×10^-6^ |
| rs12819905 | 210 | 12 | *SLC15A4* | flanking_3UTR | 0.9415 | 4.88×10^-6^ |
| rs4358672 | 211 | 6 | *LOC441179* | flanking_3UTR | 0.9415 | 4.91×10^-6^ |
| rs16842486 | 212 | 4 | *FLJ45966* | flanking_3UTR | 0.9414 | 4.91×10^-6^ |
| rs1412461 | 213 | 9 | *OSTF1* | flanking_3UTR | 0.9414 | 4.95×10^-6^ |
| rs9550757 | 214 | 13 | *FGF9* | intron | 0.9413 | 4.98×10^-6^ |
| rs10977969 | 215 | 9 | *PTPRD* | flanking_5UTR | 0.9412 | 5.00×10^-6^ |
| rs324148 | 216 | 6 | *SLC29A1* | intron | 0.9412 | 5.00×10^-6^ |
| rs234460 | 217 | 14 | *VRK1* | flanking_3UTR | 0.9411 | 5.06×10^-6^ |
| rs10006458 | 218 | 4 | *HOPX* | flanking_3UTR | 0.9411 | 5.06×10^-6^ |
| rs17461918 | 219 | 1 | *MAGI3* | intron | 0.9411 | 5.07×10^-6^ |
| rs439022 | 220 | 13 | *SLITRK5* | flanking_3UTR | 0.9411 | 5.07×10^-6^ |
| rs9777581 | 221 | 9 | *GADD45G* | flanking_3UTR | 0.9411 | 5.08×10^-6^ |
| rs1939957 | 222 | 11 | *OPCML* | intron | 0.9410 | 5.09×10^-6^ |
| rs535765 | 223 | 19 | *ZNRF4* | flanking_5UTR | 0.9410 | 5.09×10^-6^ |
| rs2482972 | 224 | 9 | *ADAMTSL1* | intron | 0.9410 | 5.10×10^-6^ |
| rs2302374 | 225 | 10 | *HABP2* | intron | 0.9409 | 5.16×10^-6^ |
| rs2606724 | 226 | 11 | *ZBTB16* | intron | 0.9408 | 5.16×10^-6^ |
| rs2269679 | 227 | 1 | *ADCY10* | intron | 0.9408 | 5.19×10^-6^ |
| rs1535 | 228 | 11 | *FADS2* | intron | 0.9408 | 5.20×10^-6^ |
| rs10492364 | 229 | 12 | *PTHLH* | intron | 0.9407 | 5.24×10^-6^ |
| rs2034233 | 230 | 5 | *STC2* | flanking_5UTR | 0.9406 | 5.26×10^-6^ |
| rs1530781 | 231 | 8 | *LZTS1* | flanking_5UTR | 0.9406 | 5.26×10^-6^ |
| rs4544728 | 232 | 4 | *ZNF827* | intron | 0.9406 | 5.26×10^-6^ |
| rs1348530 | 233 | 4 | *FSTL5* | flanking_3UTR | 0.9405 | 5.31×10^-6^ |
| rs3829 | 234 | 4 | *ZNF718* | intron | 0.9404 | 5.34×10^-6^ |
| rs2100171 | 235 | 2 | *HDAC4* | intron | 0.9404 | 5.38×10^-6^ |
| rs7514649 | 236 | 1 | *MAGI3* | intron | 0.9403 | 5.39×10^-6^ |
| rs16927632 | 237 | 12 | *SOX5* | intron | 0.9403 | 5.42×10^-6^ |
| rs4889856 | 238 | 17 | *RPTOR* | flanking_5UTR | 0.9402 | 5.43×10^-6^ |
| rs2267357 | 239 | 22 | *CACNG2* | intron | 0.9402 | 5.43×10^-6^ |
| rs2299831 | 240 | 22 | *CACNG2* | intron | 0.9402 | 5.43×10^-6^ |
| rs2267349 | 241 | 22 | *CACNG2* | intron | 0.9402 | 5.43×10^-6^ |
| rs7562270 | 242 | 2 | *NRXN1* | flanking_3UTR | 0.9402 | 5.43×10^-6^ |
| rs16972092 | 243 | 16 | *C16orf47* | flanking_5UTR | 0.9402 | 5.45×10^-6^ |
| rs13429913 | 244 | 2 | *COL4A3* | intron | 0.9401 | 5.49×10^-6^ |
| rs10506068 | 245 | 12 | *IPO8* | flanking_3UTR | 0.9400 | 5.53×10^-6^ |
| rs1438483 | 246 | X | *FRMPD4* | intron | 0.9400 | 5.55×10^-6^ |
| rs2720723 | 247 | 8 | *NEF3* | flanking_5UTR | 0.9399 | 5.59×10^-6^ |
| rs1401483 | 248 | 8 | *CSMD1* | intron | 0.9399 | 5.59×10^-6^ |
| rs11782681 | 249 | 8 | *RNF5P1* | flanking_5UTR | 0.9399 | 5.60×10^-6^ |
| rs12357700 | 250 | 10 | *COL13A1* | intron | 0.9398 | 5.61×10^-6^ |
| rs11618264 | 251 | 13 | *USPL1* | flanking_5UTR | 0.9397 | 5.65×10^-6^ |
| rs10756657 | 252 | 9 | *TTC39B* | flanking_5UTR | 0.9397 | 5.67×10^-6^ |
| rs2654189 | 253 | 17 | *SLC2A4* | flanking_5UTR | 0.9397 | 5.69×10^-6^ |
| rs1868664 | 254 | 8 | *LZTS1* | flanking_5UTR | 0.9395 | 5.76×10^-6^ |
| rs1428479 | 255 | 5 | *CD180* | flanking_5UTR | 0.9395 | 5.78×10^-6^ |
| rs17022545 | 256 | 3 | *CADM2* | flanking_5UTR | 0.9394 | 5.79×10^-6^ |
| rs6529653 | 257 | X | *MOSPD1* | flanking_5UTR | 0.9394 | 5.80×10^-6^ |
| rs6635022 | 258 | X | *MOSPD1* | flanking_5UTR | 0.9394 | 5.80×10^-6^ |
| rs1990996 | 259 | 5 | *BDP1* | flanking_5UTR | 0.9394 | 5.83×10^-6^ |
| rs4679044 | 260 | 3 | *CLASP2* | intron | 0.9393 | 5.86×10^-6^ |
| rs4780058 | 261 | 15 | *GREM1* | flanking_3UTR | 0.9392 | 5.90×10^-6^ |
| rs5750326 | 262 | 22 | *NCF4* | flanking_3UTR | 0.9392 | 5.92×10^-6^ |
| rs8032503 | 263 | 15 | *ARRDC4* | flanking_5UTR | 0.9391 | 5.93×10^-6^ |
| rs10068115 | 264 | 5 | *IRX4* | flanking_5UTR | 0.9390 | 5.98×10^-6^ |
| rs300280 | 265 | 1 | *ACOT11* | intron | 0.9390 | 5.99×10^-6^ |
| rs1437299 | 266 | 2 | *NXPH2* | flanking_5UTR | 0.9390 | 6.01×10^-6^ |
| rs4388427 | 267 | 8 | *NPAL2* | flanking_5UTR | 0.9390 | 6.02×10^-6^ |
| rs2296482 | 268 | 14 | *C14orf153* | intron | 0.9389 | 6.07×10^-6^ |
| rs12461754 | 269 | 19 | *DDX39* | coding | 0.9388 | 6.09×10^-6^ |
| rs199635 | 270 | 6 | *C6orf155* | flanking_5UTR | 0.9388 | 6.12×10^-6^ |
| rs2133483 | 271 | 13 | *KLHL1* | flanking_3UTR | 0.9386 | 6.21×10^-6^ |
| rs4806741 | 272 | 19 | *LILRA3* | flanking_5UTR | 0.9385 | 6.23×10^-6^ |
| rs4952105 | 273 | 2 | *YPEL5* | flanking_3UTR | 0.9385 | 6.23×10^-6^ |
| rs2485247 | 274 | 13 | *SLITRK6* | flanking_3UTR | 0.9385 | 6.24×10^-6^ |
| rs1434084 | 275 | 2 | *OSBPL6* | intron | 0.9385 | 6.24×10^-6^ |
| rs12294763 | 276 | 11 | *COMMD9* | flanking_5UTR | 0.9385 | 6.26×10^-6^ |
| rs4894514 | 277 | 3 | *PLD1* | flanking_5UTR | 0.9385 | 6.26×10^-6^ |
| rs11872992 | 278 | 18 | *MC4R* | flanking_5UTR | 0.9384 | 6.31×10^-6^ |
| rs9275184 | 279 | 6 | *HLA-DQB1* | flanking_5UTR | 0.9383 | 6.33×10^-6^ |
| rs6126166 | 280 | 20 | *KCNG1* | flanking_5UTR | 0.9383 | 6.35×10^-6^ |
| rs11110478 | 281 | 12 | *GAS2L3* | flanking_3UTR | 0.9382 | 6.40×10^-6^ |
| rs4805218 | 282 | 19 | *UQCRFS1* | flanking_3UTR | 0.9381 | 6.43×10^-6^ |
| rs1029225 | 283 | 21 | *CLIC6* | flanking_3UTR | 0.9381 | 6.46×10^-6^ |
| rs9681094 | 284 | 3 | *CPNE4* | flanking_5UTR | 0.9380 | 6.48×10^-6^ |
| rs11716867 | 285 | 3 | *CACNA2D3* | intron | 0.9380 | 6.48×10^-6^ |
| rs1491370 | 286 | 4 | *KCNIP4* | intron | 0.9380 | 6.49×10^-6^ |
| rs3957148 | 287 | 6 | *HLA-DQA2* | flanking_5UTR | 0.9380 | 6.50×10^-6^ |
| rs868688 | 288 | 1 | *PRDM16* | intron | 0.9380 | 6.51×10^-6^ |
| rs12056093 | 289 | 7 | *AUTS2* | intron | 0.9379 | 6.55×10^-6^ |
| rs12530267 | 290 | 6 | *DTNBP1* | flanking_5UTR | 0.9378 | 6.59×10^-6^ |
| rs12594922 | 291 | 15 | *TMCO5* | flanking_5UTR | 0.9378 | 6.6×10^-6^ |
| rs2072797 | 292 | 22 | *UNC84B* | coding | 0.9378 | 6.62×10^-6^ |
| rs12702671 | 293 | 7 | *GLCCI1* | flanking_5UTR | 0.9377 | 6.66×10^-6^ |
| rs12810080 | 294 | 12 | *RBM19* | flanking_5UTR | 0.9377 | 6.67×10^-6^ |
| rs4101508 | 295 | 3 | *KCNH8* | flanking_5UTR | 0.9375 | 6.74×10^-6^ |
| rs4900000 | 296 | 14 | *FOXN3* | flanking_5UTR | 0.9375 | 6.75×10^-6^ |
| rs4894559 | 297 | 3 | *TNFSF10* | intron | 0.9375 | 6.75×10^-6^ |
| rs547175 | 298 | 6 | *UTRN* | flanking_3UTR | 0.9375 | 6.76×10^-6^ |
| rs16823913 | 299 | 2 | *CCL20* | flanking_5UTR | 0.9374 | 6.80×10^-6^ |
| rs16869739 | 300 | 4 | *SLIT2* | intron | 0.9373 | 6.84×10^-6^ |
| rs12446955 | 301 | 16 | *MLYCD* | flanking_5UTR | 0.9373 | 6.86×10^-6^ |
| rs2204943 | 302 | 14 | *TRAV14DV4* | flanking_3UTR | 0.9372 | 6.91×10^-6^ |
| rs9796292 | 303 | 13 | *RASA3* | flanking_3UTR | 0.9372 | 6.93×10^-6^ |
| rs7148403 | 304 | 14 | *SEC23A* | flanking_3UTR | 0.9372 | 6.94×10^-6^ |
| rs9583067 | 305 | 13 | *SLITRK5* | flanking_3UTR | 0.9371 | 7.00×10^-6^ |
| rs1074199 | 306 | 13 | *SLITRK5* | flanking_3UTR | 0.9370 | 7.05×10^-6^ |
| rs7875184 | 307 | 9 | *NTRK2* | intron | 0.9369 | 7.06×10^-6^ |
| rs370526 | 308 | 3 | *SRGAP3* | intron | 0.9369 | 7.10×10^-6^ |
| rs12512351 | 309 | 4 | *RHOH* | flanking_3UTR | 0.9369 | 7.10×10^-6^ |
| rs174556 | 310 | 11 | *FADS1* | intron | 0.9369 | 7.10×10^-6^ |
| rs2344953 | 311 | 3 | *C3orf38* | flanking_3UTR | 0.9366 | 7.26×10^-6^ |
| rs37794 | 312 | 5 | *FST* | flanking_5UTR | 0.9365 | 7.28×10^-6^ |
| rs10519487 | 313 | 5 | *SEMA6A* | flanking_5UTR | 0.9365 | 7.30×10^-6^ |
| rs10022745 | 314 | 4 | *BTC* | flanking_3UTR | 0.9365 | 7.32×10^-6^ |
| rs819032 | 315 | 2 | *KIAA1715* | intron | 0.9365 | 7.32×10^-6^ |
| rs12598715 | 316 | 16 | *DYNLRB2* | flanking_5UTR | 0.9365 | 7.33×10^-6^ |
| rs174546 | 317 | 11 | *FADS1* | 3UTR | 0.9364 | 7.37×10^-6^ |
| rs4801457 | 318 | 19 | *ZNF272* | intron | 0.9364 | 7.37×10^-6^ |
| rs1232783 | 319 | 20 | *PLCB1* | intron | 0.9364 | 7.38×10^-6^ |
| rs1250550 | 320 | 10 | *ZMIZ1* | intron | 0.9364 | 7.39×10^-6^ |
| rs635754 | 321 | 1 | *PTBP2* | flanking_5UTR | 0.9362 | 7.46×10^-6^ |
| rs2052037 | 322 | 19 | *UQCRFS1* | flanking_3UTR | 0.9362 | 7.49×10^-6^ |
| rs4352181 | 323 | 2 | *LRP1B* | intron | 0.9361 | 7.52×10^-6^ |
| rs10871579 | 324 | 18 | *LINC00305* | flanking_5UTR | 0.9360 | 7.61×10^-6^ |
| rs10914271 | 325 | 1 | *PTPRU* | flanking_3UTR | 0.9360 | 7.61×10^-6^ |
| rs17639812 | 326 | 3 | *LARS2* | intron | 0.9359 | 7.65×10^-6^ |
| rs7823813 | 327 | 8 | *SCARA3* | flanking_3UTR | 0.9359 | 7.66×10^-6^ |
| rs1341483 | 328 | 13 | *SPG20* | flanking_5UTR | 0.9358 | 7.71×10^-6^ |
| rs898716 | 329 | 10 | *FRMD4A* | intron | 0.9358 | 7.73×10^-6^ |
| rs10885145 | 330 | 10 | *ADRA2A* | flanking_3UTR | 0.9357 | 7.76×10^-6^ |
| rs6648380 | 331 | X | *CHIC1* | flanking_3UTR | 0.9357 | 7.77×10^-6^ |
| rs4534896 | 332 | 17 | *SKAP1* | intron | 0.9357 | 7.77×10^-6^ |
| rs8094855 | 333 | 18 | *CBLN2* | flanking_3UTR | 0.9357 | 7.77×10^-6^ |
| rs4822502 | 334 | 22 | *UPB1* | flanking_5UTR | 0.9356 | 7.84×10^-6^ |
| rs13203409 | 335 | 6 | *LOC441179* | flanking_3UTR | 0.9356 | 7.85×10^-6^ |
| rs11975075 | 336 | 7 | *POM121L12* | flanking_5UTR | 0.9355 | 7.88×10^-6^ |
| rs7228283 | 337 | 18 | *DSEL* | flanking_3UTR | 0.9355 | 7.91×10^-6^ |
| rs12128782 | 338 | 1 | *TBX19* | flanking_3UTR | 0.9355 | 7.91×10^-6^ |
| rs9908451 | 339 | 17 | *HS3ST3A1* | flanking_5UTR | 0.9354 | 7.92×10^-6^ |
| rs1458001 | 340 | 3 | *C3orf38* | flanking_3UTR | 0.9354 | 7.93×10^-6^ |
| rs2831057 | 341 | 21 | *LINC00314* | flanking_5UTR | 0.9354 | 7.95×10^-6^ |
| rs7623768 | 342 | 3 | *CRTAP* | intron | 0.9354 | 7.96×10^-6^ |
| rs10022199 | 343 | 4 | *BTC* | flanking_3UTR | 0.9354 | 7.97×10^-6^ |
| rs1843242 | 344 | 12 | *SLC15A4* | flanking_3UTR | 0.9353 | 8.02×10^-6^ |
| rs7970148 | 345 | 12 | *SLC15A4* | flanking_3UTR | 0.9352 | 8.05×10^-6^ |
| rs1843240 | 346 | 12 | *SLC15A4* | flanking_3UTR | 0.9352 | 8.05×10^-6^ |
| rs4144407 | 347 | 18 | *SYT4* | flanking_5UTR | 0.9352 | 8.07×10^-6^ |
| rs34569 | 348 | 5 | *EFNA5* | flanking_3UTR | 0.9352 | 8.08×10^-6^ |
| rs1957015 | 349 | 14 | *AKAP6* | intron | 0.9351 | 8.10×10^-6^ |
| rs2192703 | 350 | 2 | *THUMPD2* | flanking_5UTR | 0.9351 | 8.11×10^-6^ |
| rs16763 | 351 | 9 | *VAV2* | intron | 0.9351 | 8.13×10^-6^ |
| rs760703 | 352 | 20 | *PTPRT* | intron | 0.9351 | 8.13×10^-6^ |
| rs10843505 | 353 | 12 | *TMTC1* | intron | 0.9351 | 8.15×10^-6^ |
| rs854889 | 354 | 12 | *AMIGO2* | 3UTR | 0.9350 | 8.17×10^-6^ |
| rs2242226 | 355 | 4 | *TRIML1* | flanking_3UTR | 0.9350 | 8.21×10^-6^ |
| rs10177490 | 356 | 2 | *EN1* | flanking_3UTR | 0.9349 | 8.23×10^-6^ |
| rs11968687 | 357 | 6 | *OGFRL1* | intron | 0.9349 | 8.24×10^-6^ |
| rs12495929 | 358 | 3 | *SUCNR1* | flanking_3UTR | 0.9349 | 8.25×10^-6^ |
| rs1530357 | 359 | 17 | *HS3ST3A1* | flanking_5UTR | 0.9348 | 8.33×10^-6^ |
| rs7217869 | 360 | 17 | *TEKT3* | flanking_5UTR | 0.9347 | 8.35×10^-6^ |
| rs12284877 | 361 | 11 | *GUCY1A2* | flanking_3UTR | 0.9347 | 8.35×10^-6^ |
| rs2658941 | 362 | 8 | *XKR4* | intron | 0.9347 | 8.37×10^-6^ |
| rs2622546 | 363 | 8 | *XKR4* | intron | 0.9347 | 8.37×10^-6^ |
| rs1513029 | 364 | 13 | *SLC10A2* | flanking_5UTR | 0.9346 | 8.42×10^-6^ |
| rs1020684 | 365 | 13 | *SLITRK5* | flanking_3UTR | 0.9345 | 8.50×10^-6^ |
| rs998107 | 366 | 17 | *MMD* | flanking_5UTR | 0.9343 | 8.66×10^-6^ |
| rs4779521 | 367 | 15 | *KLF13* | flanking_3UTR | 0.9342 | 8.70×10^-6^ |
| rs10135529 | 368 | 14 | *FLRT2* | flanking_5UTR | 0.9341 | 8.75×10^-6^ |
| rs7100599 | 369 | 10 | *PBLD* | flanking_5UTR | 0.9340 | 8.79×10^-6^ |
| rs11629363 | 370 | 14 | *DACT1* | flanking_3UTR | 0.9340 | 8.84×10^-6^ |
| rs657672 | 371 | 17 | *CACNB1* | flanking_3UTR | 0.9340 | 8.85×10^-6^ |
| rs1352388 | 372 | 12 | *ITPR2* | intron | 0.9339 | 8.86×10^-6^ |
| rs12613347 | 373 | 2 | *CASP10* | intron | 0.9338 | 8.96×10^-6^ |
| rs10174976 | 374 | 2 | *CRIM1* | flanking_5UTR | 0.9337 | 9.00×10^-6^ |
| rs2271690 | 375 | 10 | *SAR1A* | intron | 0.9336 | 9.06×10^-6^ |
| rs9310457 | 376 | 3 | *CCDC174* | flanking_5UTR | 0.9335 | 9.12×10^-6^ |
| rs12879663 | 377 | 14 | *MARK3* | intron | 0.9335 | 9.12×10^-6^ |
| rs317969 | 378 | 5 | *CD180* | flanking_5UTR | 0.9335 | 9.14×10^-6^ |
| rs1208932 | 379 | 1 | *LUZP1* | intron | 0.9335 | 9.14×10^-6^ |
| rs2965015 | 380 | 1 | *TGFB2* | flanking_3UTR | 0.9335 | 9.16×10^-6^ |
| rs2022345 | 381 | 6 | *DTNBP1* | flanking_5UTR | 0.9335 | 9.16×10^-6^ |
| rs2304693 | 382 | 7 | *TBRG4* | coding | 0.9334 | 9.20×10^-6^ |
| rs2304694 | 383 | 7 | *TBRG4* | coding | 0.9334 | 9.20×10^-6^ |
| rs2735202 | 384 | 11 | *ZBTB16* | intron | 0.9334 | 9.20×10^-6^ |
| rs642162 | 385 | 6 | *CITED2* | flanking_5UTR | 0.9334 | 9.20×10^-6^ |
| rs9299422 | 386 | 9 | *HIATL1* | intron | 0.9334 | 9.21×10^-6^ |
| rs10823440 | 387 | 10 | *COL13A1* | intron | 0.9334 | 9.22×10^-6^ |
| rs2076962 | 388 | 16 | *FBXO31* | flanking_3UTR | 0.9333 | 9.31×10^-6^ |
| rs11131685 | 389 | 4 | *CENPC1* | flanking_3UTR | 0.9332 | 9.35×10^-6^ |
| rs2226441 | 390 | 21 | *NCAM2* | flanking_3UTR | 0.9331 | 9.39×10^-6^ |
| rs12884068 | 391 | 14 | *MAP3K9* | flanking_5UTR | 0.9330 | 9.50×10^-6^ |
| rs12525101 | 392 | 6 | *FARS2* | intron | 0.9330 | 9.50×10^-6^ |
| rs884940 | 393 | 1 | *SKI* | intron | 0.9330 | 9.51×10^-6^ |
| rs7170529 | 394 | 15 | *LRRC28* | flanking_3UTR | 0.9330 | 9.51×10^-6^ |
| rs12172202 | 395 | 22 | *DMC1* | intron | 0.9330 | 9.52×10^-6^ |
| rs185634 | 396 | 6 | *C6orf155* | flanking_5UTR | 0.9330 | 9.52×10^-6^ |
| rs10942089 | 397 | 5 | *CDH10* | flanking_5UTR | 0.9329 | 9.57×10^-6^ |
| rs630902 | 398 | 4 | *PPARGC1A* | flanking_5UTR | 0.9328 | 9.61×10^-6^ |
| rs12695988 | 399 | 3 | *RAP2B* | flanking_3UTR | 0.9328 | 9.62×10^-6^ |
| rs4373430 | 400 | 7 | *TRGV2* | flanking_5UTR | 0.9328 | 9.65×10^-6^ |
| rs1400028 | 401 | 2 | *FAM84A* | flanking_5UTR | 0.9327 | 9.68×10^-6^ |

*Note*: CAT, climatic ambient temperature. Chr, chromosome.
