# Supplementary material for: Ambient Temperature is A Strong Selective Factor Influencing Human Development and Immunity
Source: Genomics Proteomics Bioinformatics. 2020 Aug 19;18(5):489–500. doi: 10.1016/j.gpb.2019.11.009 (PMC8377383; doi:10.1016/j.gpb.2019.11.009)
Supplement: Supplementary Table S2 [file mmc2.doc]

**Table S2 UVR-associated SNPs at the suggestive 1×10^-5^** level

| **Name** | **Rank** | **Chr** | **Gene symbol** | **Location** | ***r*** | ***P*** |
| --- | --- | --- | --- | --- | --- | --- |
| rs7531583 | 1 | 1 | *NADK* | intron | 0.9740 | 8.90×10^-8^ |
| rs1586360 | 2 | 7 | *IGFBP3* | flanking_5UTR | 0.9734 | 9.96×10^-8^ |
| rs10181642 | 3 | 2 | *KIAA1715* | flanking_5UTR | 0.9717 | 1.37×10^-7^ |
| rs6755912 | 4 | 2 | *SPAG16* | intron | 0.9715 | 1.42×10^-7^ |
| rs3816186 | 5 | 2 | *MTA3* | flanking_3UTR | 0.9714 | 1.43×10^-7^ |
| rs941736 | 6 | 20 | *RIN2* | flanking_5UTR | 0.9698 | 1.89×10^-7^ |
| rs6795768 | 7 | 3 | *FNDC3B* | intron | 0.9667 | 3.07×10^-7^ |
| rs385771 | 8 | 5 | *THBS4* | flanking_5UTR | 0.9664 | 3.18×10^-7^ |
| rs7793301 | 9 | 7 | *CRYGN* | flanking_3UTR | 0.9661 | 3.35×10^-7^ |
| rs10499198 | 10 | 6 | *TNFAIP3* | flanking_3UTR | 0.9659 | 3.44×10^-7^ |
| rs7185008 | 11 | 16 | *MAF* | flanking_5UTR | 0.9626 | 5.43×10^-7^ |
| rs1861523 | 12 | X | *GLUD2* | flanking_3UTR | 0.9614 | 6.36×10^-7^ |
| rs9285647 | 13 | 5 | *NMUR2* | flanking_5UTR | 0.9588 | 8.69×10^-7^ |
| rs7565081 | 14 | 2 | *ALS2CR7* | flanking_3UTR | 0.9572 | 1.05×10^-6^ |
| rs17364223 | 15 | 7 | *KIAA0241* | intron | 0.9568 | 1.10×10^-6^ |
| rs2825305 | 16 | 21 | *PRSS7* | flanking_5UTR | 0.9563 | 1.17×10^-6^ |
| rs10515027 | 17 | 17 | *CA10* | flanking_5UTR | 0.9561 | 1.19×10^-6^ |
| rs2207216 | 18 | 1 | *DPT* | intron | 0.9555 | 1.28×10^-6^ |
| rs1953192 | 19 | 14 | *FBXO33* | flanking_5UTR | 0.9550 | 1.35×10^-6^ |
| rs3011468 | 20 | 13 | *MCF2L* | flanking_5UTR | 0.9547 | 1.39×10^-6^ |
| rs1227079 | 21 | 10 | *CDH23* | intron | 0.9545 | 1.42×10^-6^ |
| rs4521180 | 22 | 3 | *RBMS3* | flanking_3UTR | 0.9538 | 1.54×10^-6^ |
| rs12896363 | 23 | 14 | *TCL1A* | flanking_5UTR | 0.9534 | 1.60×10^-6^ |
| rs4586843 | 24 | 3 | *FNDC3B* | intron | 0.9534 | 1.60×10^-6^ |
| rs6663915 | 25 | 1 | *PIN1L* | flanking_5UTR | 0.9534 | 1.60×10^-6^ |
| rs2052070 | 26 | 10 | *LYZL1* | flanking_5UTR | 0.9534 | 1.60×10^-6^ |
| rs1957210 | 27 | 14 | *FBXO33* | flanking_5UTR | 0.9533 | 1.61×10^-6^ |
| rs11130036 | 28 | 3 | *ITPR1* | intron | 0.9533 | 1.62×10^-6^ |
| rs2582843 | 29 | 8 | *SDC2* | intron | 0.9528 | 1.70×10^-6^ |
| rs10130134 | 30 | 14 | *C14orf159* | intron | 0.9525 | 1.75×10^-6^ |
| rs490262 | 31 | 11 | *CEP164* | coding | 0.9522 | 1.81×10^-6^ |
| rs12536153 | 32 | 7 | *LOC154907* | flanking_5UTR | 0.9521 | 1.83×10^-6^ |
| rs7586691 | 33 | 2 | *CRIM1* | flanking_5UTR | 0.9520 | 1.86×10^-6^ |
| rs2052037 | 34 | 19 | *UQCRFS1* | flanking_3UTR | 0.9518 | 1.88×10^-6^ |
| rs10494989 | 35 | 1 | *KCNK2* | intron | 0.9518 | 1.88×10^-6^ |
| rs11577496 | 36 | 1 | *ACTL8* | intron | 0.9517 | 1.90×10^-6^ |
| rs998107 | 37 | 17 | *MMD* | flanking_5UTR | 0.9517 | 1.90×10^-6^ |
| rs9664222 | 38 | 10 | *MINPP1* | flanking_3UTR | 0.9516 | 1.92×10^-6^ |
| rs7652889 | 39 | 3 | *BFSP2* | flanking_3UTR | 0.9514 | 1.96×10^-6^ |
| rs2601607 | 40 | 1 | *KCNK2* | intron | 0.9513 | 1.98×10^-6^ |
| rs10878661 | 41 | 12 | *CNTN1* | flanking_5UTR | 0.9509 | 2.07×10^-6^ |
| rs2715520 | 42 | 4 | *AGA* | flanking_5UTR | 0.9507 | 2.12×10^-6^ |
| rs6573229 | 43 | 14 | *DAAM1* | flanking_5UTR | 0.9506 | 2.13×10^-6^ |
| rs557668 | 44 | 21 | *CLDN17* | flanking_3UTR | 0.9505 | 2.16×10^-6^ |
| rs10829321 | 45 | 10 | *PTPRE* | intron | 0.9504 | 2.17×10^-6^ |
| rs2340713 | 46 | 16 | *NAGPA* | flanking_3UTR | 0.9500 | 2.26×10^-6^ |
| rs11197596 | 47 | 10 | *GFRA1* | intron | 0.9496 | 2.35×10^-6^ |
| rs2043497 | 48 | 7 | *PTPRN2* | intron | 0.9495 | 2.37×10^-6^ |
| rs2415562 | 49 | 14 | *FBXO33* | flanking_5UTR | 0.9491 | 2.47×10^-6^ |
| rs10432228 | 50 | 18 | *CD226* | intron | 0.9488 | 2.53×10^-6^ |
| rs256962 | 51 | 5 | *TMED7* | flanking_3UTR | 0.9486 | 2.60×10^-6^ |
| rs10502699 | 52 | 18 | *BRUNOL4* | flanking_5UTR | 0.9484 | 2.63×10^-6^ |
| rs305000 | 53 | 15 | *TLE3* | flanking_3UTR | 0.9483 | 2.67×10^-6^ |
| rs4395226 | 54 | 2 | *RAPGEF4* | flanking_5UTR | 0.9482 | 2.69×10^-6^ |
| rs6044262 | 55 | 20 | *SNRPB2* | flanking_5UTR | 0.9482 | 2.70×10^-6^ |
| rs4889545 | 56 | 16 | *C16orf58* | flanking_5UTR | 0.9478 | 2.80×10^-6^ |
| rs2114824 | 57 | 10 | *GRID1* | intron | 0.9477 | 2.81×10^-6^ |
| rs12565228 | 58 | 1 | *GRRP1* | flanking_5UTR | 0.9477 | 2.83×10^-6^ |
| rs4472861 | 59 | 10 | *XPNPEP1* | flanking_3UTR | 0.9476 | 2.85×10^-6^ |
| rs7907322 | 60 | 10 | *CXXC6* | intron | 0.9475 | 2.88×10^-6^ |
| rs4652143 | 61 | 1 | *RFWD2* | intron | 0.9475 | 2.88×10^-6^ |
| rs1691018 | 62 | 5 | *IRX1* | flanking_5UTR | 0.9474 | 2.90×10^-6^ |
| rs1432218 | 63 | 2 | *HNMT* | flanking_5UTR | 0.9471 | 2.99×10^-6^ |
| rs857591 | 64 | 1 | *PRG4* | flanking_5UTR | 0.9470 | 3.01×10^-6^ |
| rs325793 | 65 | 3 | *MBNL1* | flanking_5UTR | 0.9467 | 3.09×10^-6^ |
| rs12623537 | 66 | 2 | *SSFA2* | flanking_3UTR | 0.9466 | 3.12×10^-6^ |
| rs1001099 | 67 | 7 | *PTPRN2* | intron | 0.9465 | 3.16×10^-6^ |
| rs7858547 | 68 | 9 | *SLC24A2* | flanking_3UTR | 0.9462 | 3.25×10^-6^ |
| rs1524594 | 69 | 7 | *C7orf25* | flanking_3UTR | 0.9459 | 3.32×10^-6^ |
| rs4829222 | 70 | X | *DMD* | intron | 0.9457 | 3.39×10^-6^ |
| rs10433639 | 71 | 3 | *PTPRG* | intron | 0.9456 | 3.43×10^-6^ |
| rs2832587 | 72 | 21 | *CLDN17* | flanking_3UTR | 0.9454 | 3.47×10^-6^ |
| rs6546281 | 73 | 2 | *ETAA16* | flanking_5UTR | 0.9453 | 3.52×10^-6^ |
| rs16857230 | 74 | 4 | *YIPF7* | flanking_3UTR | 0.9452 | 3.54×10^-6^ |
| rs10861534 | 75 | 12 | *NUAK1* | flanking_3UTR | 0.9451 | 3.57×10^-6^ |
| rs726697 | 76 | 13 | *EFNB2* | flanking_3UTR | 0.9451 | 3.57×10^-6^ |
| rs4341514 | 77 | 11 | *C11orf43* | flanking_3UTR | 0.9451 | 3.57×10^-6^ |
| rs9386196 | 78 | 6 | *LOC389432* | flanking_3UTR | 0.9448 | 3.68×10^-6^ |
| rs11117495 | 79 | 16 | *FOXF1* | flanking_5UTR | 0.9447 | 3.71×10^-6^ |
| rs4762002 | 80 | 12 | *SCN8A* | intron | 0.9446 | 3.73×10^-6^ |
| rs874961 | 81 | 22 | *LOC388915* | flanking_5UTR | 0.9445 | 3.78×10^-6^ |
| rs1331885 | 82 | 9 | *LRRN6C* | intron | 0.9440 | 3.95×10^-6^ |
| rs10103568 | 83 | 8 | *WDR21C* | flanking_3UTR | 0.9438 | 4.02×10^-6^ |
| rs11110478 | 84 | 12 | *GAS2L3* | flanking_3UTR | 0.9437 | 4.04×10^-6^ |
| rs339446 | 85 | 5 | *DNAH5* | intron | 0.9436 | 4.07×10^-6^ |
| rs10878626 | 86 | 12 | *CNTN1* | flanking_5UTR | 0.9436 | 4.08×10^-6^ |
| rs2588400 | 87 | 12 | *CNTN1* | flanking_5UTR | 0.9436 | 4.08×10^-6^ |
| rs10139267 | 88 | 14 | *FBXO33* | flanking_5UTR | 0.9436 | 4.08×10^-6^ |
| rs2924685 | 89 | 11 | *CPT1A* | intron | 0.9433 | 4.20×10^-6^ |
| rs2061459 | 90 | 4 | *EPHA5* | flanking_5UTR | 0.9432 | 4.22×10^-6^ |
| rs973406 | 91 | 2 | *MTX2* | flanking_5UTR | 0.9432 | 4.23×10^-6^ |
| rs10430083 | 92 | 1 | *PRMT6* | flanking_5UTR | 0.9431 | 4.26×10^-6^ |
| rs1838039 | 93 | 4 | *WDFY3* | intron | 0.9430 | 4.31×10^-6^ |
| rs716365 | 94 | 11 | *LOC119710* | flanking_3UTR | 0.9430 | 4.31×10^-6^ |
| rs4696584 | 95 | 4 | *DCHS2* | intron | 0.9429 | 4.33×10^-6^ |
| rs942699 | 96 | 1 | *ST6GALNAC3* | intron | 0.9427 | 4.41×10^-6^ |
| rs39897 | 97 | 5 | *CSF2* | flanking_3UTR | 0.9426 | 4.44×10^-6^ |
| rs4404733 | 98 | 5 | *NMUR2* | flanking_5UTR | 0.9424 | 4.52×10^-6^ |
| rs1544131 | 99 | 1 | *HSPB7* | flanking_5UTR | 0.9424 | 4.53×10^-6^ |
| rs2657902 | 100 | 12 | *RBMS2* | intron | 0.9422 | 4.62×10^-6^ |
| rs1322142 | 101 | 9 | *PTPRD* | flanking_5UTR | 0.9422 | 4.62×10^-6^ |
| rs11247870 | 102 | 1 | *CATSPER4* | intron | 0.9421 | 4.65×10^-6^ |
| rs10155664 | 103 | 6 | *ID4* | flanking_5UTR | 0.9420 | 4.67×10^-6^ |
| rs868688 | 104 | 1 | *PRDM16* | intron | 0.9419 | 4.74×10^-6^ |
| rs9524157 | 105 | 13 | *GPC6* | intron | 0.9417 | 4.82×10^-6^ |
| rs446170 | 106 | 16 | *CDH5* | flanking_5UTR | 0.9416 | 4.85×10^-6^ |
| rs6782714 | 107 | 3 | *EIF4E3* | flanking_3UTR | 0.9416 | 4.86×10^-6^ |
| rs10788027 | 108 | 10 | *SEC23IP* | flanking_3UTR | 0.9414 | 4.93×10^-6^ |
| rs7247342 | 109 | 19 | *UQCRFS1* | flanking_3UTR | 0.9413 | 4.99×10^-6^ |
| rs10506375 | 110 | 12 | *LRIG3* | flanking_3UTR | 0.9412 | 5.03×10^-6^ |
| rs12467466 | 111 | 2 | *CENPA* | intron | 0.9411 | 5.06×10^-6^ |
| rs1554245 | 112 | 4 | *PROM1* | flanking_5UTR | 0.9409 | 5.12×10^-6^ |
| rs10998304 | 113 | 10 | *CXXC6* | intron | 0.9408 | 5.20×10^-6^ |
| rs1446040 | 114 | 5 | *ANKH* | flanking_5UTR | 0.9407 | 5.21×10^-6^ |
| rs7030322 | 115 | 9 | *LOC497256* | flanking_5UTR | 0.9407 | 5.22×10^-6^ |
| rs7620000 | 116 | 3 | *SPATA16* | flanking_5UTR | 0.9406 | 5.27×10^-6^ |
| rs7269423 | 117 | 20 | *SNRPB2* | flanking_5UTR | 0.9405 | 5.30×10^-6^ |
| rs235130 | 118 | 16 | *CDH5* | flanking_5UTR | 0.9405 | 5.31×10^-6^ |
| rs2143081 | 119 | 6 | *TFAP2B* | flanking_5UTR | 0.9399 | 5.58×10^-6^ |
| rs38321 | 120 | 7 | *AUTS2* | intron | 0.9394 | 5.81×10^-6^ |
| rs3017366 | 121 | 18 | *TNFRSF11A* | flanking_3UTR | 0.9392 | 5.89×10^-6^ |
| rs7222434 | 122 | 17 | *GAS7* | intron | 0.9390 | 5.98×10^-6^ |
| rs2841612 | 123 | 1 | *KCNK2* | intron | 0.9390 | 5.99×10^-6^ |
| rs4816043 | 124 | 20 | *TXNDC13* | flanking_5UTR | 0.9390 | 6.01×10^-6^ |
| rs1349250 | 125 | 14 | *LRFN5* | flanking_3UTR | 0.9390 | 6.02×10^-6^ |
| rs10068115 | 126 | 5 | *IRX4* | flanking_5UTR | 0.9389 | 6.03×10^-6^ |
| rs2180311 | 127 | 1 | *GNB1* | intron | 0.9389 | 6.06×10^-6^ |
| rs9366309 | 128 | 6 | *ID4* | flanking_5UTR | 0.9389 | 6.07×10^-6^ |
| rs11130110 | 129 | 3 | *CCDC12* | intron | 0.9387 | 6.13×10^-6^ |
| rs2657888 | 130 | 12 | *RBMS2* | intron | 0.9385 | 6.26×10^-6^ |
| rs1542114 | 131 | 8 | *PNMA2* | flanking_5UTR | 0.9385 | 6.27×10^-6^ |
| rs10492949 | 132 | 1 | *NPHP4* | flanking_3UTR | 0.9382 | 6.40×10^-6^ |
| rs10899444 | 133 | 11 | *SHANK2* | flanking_5UTR | 0.9382 | 6.40×10^-6^ |
| rs2098383 | 134 | 2 | *FLJ13391* | flanking_3UTR | 0.9382 | 6.42×10^-6^ |
| rs7068072 | 135 | 10 | *HIF1AN* | flanking_3UTR | 0.9380 | 6.50×10^-6^ |
| rs1687626 | 136 | 16 | *FOXF1* | flanking_5UTR | 0.9379 | 6.53×10^-6^ |
| rs2291188 | 137 | 2 | *HDAC4* | intron | 0.9379 | 6.54×10^-6^ |
| rs32680 | 138 | 5 | *PAM* | flanking_5UTR | 0.9379 | 6.57×10^-6^ |
| rs9525253 | 139 | 13 | *RASA3* | intron | 0.9379 | 6.57×10^-6^ |
| rs1226473 | 140 | 6 | *GPR110* | intron | 0.9378 | 6.60×10^-6^ |
| rs7831925 | 141 | 8 | *LOC138046* | flanking_5UTR | 0.9376 | 6.71×10^-6^ |
| rs10440995 | 142 | 7 | *PHF14* | flanking_3UTR | 0.9376 | 6.72×10^-6^ |
| rs661697 | 143 | 10 | *CXCL12* | flanking_3UTR | 0.9374 | 6.81×10^-6^ |
| rs854685 | 144 | 17 | *CCL14* | flanking_5UTR | 0.9374 | 6.82×10^-6^ |
| rs11201985 | 145 | 10 | *GRID1* | intron | 0.9374 | 6.83×10^-6^ |
| rs4857882 | 146 | 3 | *KLHDC6* | intron | 0.9373 | 6.84×10^-6^ |
| rs7844372 | 147 | 8 | *CSMD3* | flanking_5UTR | 0.9373 | 6.86×10^-6^ |
| rs10844563 | 148 | 12 | *SYT10* | flanking_3UTR | 0.9372 | 6.92×10^-6^ |
| rs7778566 | 149 | 7 | *GeneID:28610* | flanking_3UTR | 0.9372 | 6.93×10^-6^ |
| rs960479 | 150 | 21 | *SAMSN1* | flanking_5UTR | 0.9369 | 7.07×10^-6^ |
| rs12272290 | 151 | 11 | *C11orf39* | flanking_3UTR | 0.9369 | 7.09×10^-6^ |
| rs7821187 | 152 | 8 | *PXDNL* | flanking_3UTR | 0.9368 | 7.14×10^-6^ |
| rs13257991 | 153 | 8 | *CSMD3* | flanking_5UTR | 0.9367 | 7.20×10^-6^ |
| rs720780 | 154 | 7 | *DYNC1I1* | intron | 0.9364 | 7.38×10^-6^ |
| rs12151388 | 155 | 2 | *MTX2* | intron | 0.9362 | 7.50×10^-6^ |
| rs6425609 | 156 | 1 | *CEP350* | flanking_3UTR | 0.9360 | 7.60×10^-6^ |
| rs305002 | 157 | 15 | *TLE3* | flanking_3UTR | 0.9359 | 7.63×10^-6^ |
| rs2579503 | 158 | 2 | *ARID5A* | flanking_5UTR | 0.9358 | 7.71×10^-6^ |
| rs10510632 | 159 | 3 | *RBMS3* | flanking_3UTR | 0.9356 | 7.82×10^-6^ |
| rs2392829 | 160 | 8 | *PXDNL* | flanking_3UTR | 0.9355 | 7.88×10^-6^ |
| rs4747574 | 161 | 10 | *ITIH2* | intron | 0.9354 | 7.94×10^-6^ |
| rs2128001 | 162 | 8 | *SNTG1* | intron | 0.9353 | 8.00×10^-6^ |
| rs6934597 | 163 | 6 | *FRK* | flanking_3UTR | 0.9352 | 8.04×10^-6^ |
| rs2976537 | 164 | 8 | *NDRG1* | flanking_5UTR | 0.9352 | 8.06×10^-6^ |
| rs2195914 | 165 | 3 | *KLHDC6* | intron | 0.9352 | 8.09×10^-6^ |
| rs3909148 | 166 | 7 | *POMZP3* | flanking_5UTR | 0.9351 | 8.13×10^-6^ |
| rs10792260 | 167 | 11 | *MS4A4A* | 5UTR | 0.9351 | 8.15×10^-6^ |
| rs12115792 | 168 | 9 | *EPB41L4B* | flanking_5UTR | 0.9349 | 8.24×10^-6^ |
| rs3017368 | 169 | 18 | *TNFRSF11A* | flanking_3UTR | 0.9349 | 8.26×10^-6^ |
| rs7821078 | 170 | 8 | *CSMD3* | flanking_5UTR | 0.9348 | 8.31×10^-6^ |
| rs8006182 | 171 | 14 | *CTAGE5* | flanking_3UTR | 0.9348 | 8.34×10^-6^ |
| rs17197569 | 172 | 2 | *C2orf26* | flanking_3UTR | 0.9347 | 8.38×10^-6^ |
| rs17348914 | 173 | 8 | *ADRA1A* | flanking_5UTR | 0.9346 | 8.43×10^-6^ |
| rs12634818 | 174 | 3 | *CCNL1* | flanking_5UTR | 0.9346 | 8.46×10^-6^ |
| rs11599653 | 175 | 10 | *WAC* | flanking_5UTR | 0.9346 | 8.46×10^-6^ |
| rs2060609 | 176 | 14 | *LRFN5* | flanking_3UTR | 0.9345 | 8.48×10^-6^ |
| rs5957625 | 177 | X | *GLUD2* | flanking_3UTR | 0.9344 | 8.54×10^-6^ |
| rs1842501 | 178 | 8 | *XKR4* | intron | 0.9344 | 8.55×10^-6^ |
| rs1463140 | 179 | 8 | *TOX* | intron | 0.9344 | 8.55×10^-6^ |
| rs1320306 | 180 | 5 | *S100Z* | flanking_3UTR | 0.9344 | 8.58×10^-6^ |
| rs7255203 | 181 | 19 | *MGC10471* | flanking_5UTR | 0.9344 | 8.59×10^-6^ |
| rs1579303 | 182 | 5 | *BTNL9* | flanking_3UTR | 0.9343 | 8.63×10^-6^ |
| rs623917 | 183 | 8 | *CHD7* | flanking_5UTR | 0.9342 | 8.70×10^-6^ |
| rs7173285 | 184 | 15 | *CA12* | intron | 0.9342 | 8.71×10^-6^ |
| rs10178599 | 185 | 2 | *COL6A3* | intron | 0.9341 | 8.73×10^-6^ |
| rs2254924 | 186 | 6 | *LOC553137* | flanking_3UTR | 0.9341 | 8.74×10^-6^ |
| rs4766899 | 187 | 12 | *SUDS3* | flanking_5UTR | 0.9340 | 8.81×10^-6^ |
| rs7017060 | 188 | 8 | *TMEM66* | flanking_3UTR | 0.9340 | 8.81×10^-6^ |
| rs10093305 | 189 | 8 | *WDR21C* | flanking_3UTR | 0.9340 | 8.81×10^-6^ |
| rs7501899 | 190 | 17 | *C17orf54* | flanking_5UTR | 0.9340 | 8.82×10^-6^ |
| rs7254583 | 191 | 19 | *UQCRFS1* | flanking_3UTR | 0.9340 | 8.82×10^-6^ |
| rs793801 | 192 | 4 | *IRF2* | intron | 0.9340 | 8.84×10^-6^ |
| rs1542437 | 193 | 4 | *SPATA5* | intron | 0.9339 | 8.89×10^-6^ |
| rs12374103 | 194 | 3 | *MAGI1* | intron | 0.9339 | 8.90×10^-6^ |
| rs2025896 | 195 | 1 | *LRRC7* | intron | 0.9339 | 8.91×10^-6^ |
| rs2427340 | 196 | 20 | *C20orf151* | flanking_5UTR | 0.9339 | 8.92×10^-6^ |
| rs407190 | 197 | 5 | *IRX1* | flanking_5UTR | 0.9339 | 8.92×10^-6^ |
| rs896036 | 198 | 18 | *MBP* | flanking_5UTR | 0.9338 | 8.96×10^-6^ |
| rs7207955 | 199 | 17 | *MGAT5B* | intron | 0.9338 | 8.96×10^-6^ |
| rs12617873 | 200 | 2 | *TAIP-2* | intron | 0.9337 | 9.04×10^-6^ |
| rs13061071 | 201 | 3 | *HYPB* | intron | 0.9336 | 9.07×10^-6^ |
| rs7143793 | 202 | 14 | *FBXO33* | flanking_5UTR | 0.9335 | 9.13×10^-6^ |
| rs4866652 | 203 | 5 | *IRX4* | flanking_5UTR | 0.9335 | 9.14×10^-6^ |
| rs7299715 | 204 | 12 | *BHLHB3* | flanking_3UTR | 0.9335 | 9.14×10^-6^ |
| rs6656216 | 205 | 1 | *MATN1* | flanking_3UTR | 0.9335 | 9.17×10^-6^ |
| rs1776947 | 206 | 20 | *SLC23A2* | intron | 0.9335 | 9.17×10^-6^ |
| rs4939702 | 207 | 18 | *DCC* | intron | 0.9334 | 9.23×10^-6^ |
| rs6746132 | 208 | 2 | *TSN* | flanking_3UTR | 0.9334 | 9.23×10^-6^ |
| rs9369649 | 209 | 6 | *GPR110* | intron | 0.9334 | 9.23×10^-6^ |
| rs1960385 | 210 | 4 | *FLJ23191* | flanking_5UTR | 0.9332 | 9.36×10^-6^ |
| rs1109541 | 211 | 16 | *KIAA1576* | intron | 0.9331 | 9.41×10^-6^ |
| rs5959303 | 212 | X | *ITM2A* | flanking_3UTR | 0.9331 | 9.43×10^-6^ |
| rs2989868 | 213 | 1 | *FLJ10986* | flanking_5UTR | 0.9330 | 9.51×10^-6^ |
| rs570306 | 214 | 10 | *MGMT* | flanking_5UTR | 0.9330 | 9.52×10^-6^ |
| rs9545617 | 215 | 13 | *SPRY2* | flanking_5UTR | 0.9329 | 9.56×10^-6^ |
| rs4969549 | 216 | X | *ZFX* | flanking_3UTR | 0.9327 | 9.70×10^-6^ |
| rs6660744 | 217 | 1 | *CATSPER4* | intron | 0.9327 | 9.73×10^-6^ |
| rs1411189 | 218 | 10 | *WAC* | intron | 0.9326 | 9.74×10^-6^ |
| rs6481534 | 219 | 10 | *WAC* | intron | 0.9326 | 9.74×10^-6^ |
| rs382013 | 220 | 22 | *BID* | flanking_5UTR | 0.9326 | 9.77×10^-6^ |
| rs7670762 | 221 | 4 | *DKFZP686A01247* | intron | 0.9326 | 9.79×10^-6^ |
| rs532413 | 222 | 21 | *CLDN17* | flanking_3UTR | 0.9326 | 9.79×10^-6^ |
| rs4836453 | 223 | 5 | *ADAMTS19* | flanking_5UTR | 0.9325 | 9.82×10^-6^ |
| rs618751 | 224 | 1 | *AJAP1* | flanking_5UTR | 0.9325 | 9.88×10^-6^ |
| rs1346310 | 225 | 12 | *CNTN1* | flanking_5UTR | 0.9324 | 9.89×10^-6^ |
| rs3814115 | 226 | 9 | *PCSK5* | flanking_5UTR | 0.9324 | 9.90×10^-6^ |
| rs4508915 | 227 | 4 | *FLJ23191* | flanking_5UTR | 0.9324 | 9.90×10^-6^ |
| rs3784601 | 228 | 15 | *TRPM1* | intron | 0.9324 | 9.91×10^-6^ |
| rs7120183 | 229 | 11 | *ETS1* | flanking_3UTR | 0.9324 | 9.91×10^-6^ |
| rs4603646 | 230 | 18 | *ZNF521* | intron | 0.9324 | 9.91×10^-6^ |
| rs176649 | 231 | 15 | *RHCG* | flanking_3UTR | 0.9324 | 9.95×10^-6^ |
| rs235083 | 232 | 16 | *CDH5* | flanking_5UTR | 0.9323 | 9.96×10^-6^ |
| rs17302656 | 233 | 3 | *MINA* | flanking_3UTR | 0.9323 | 9.96×10^-6^ |

*Note*: UVR, ultraviolet radiation. Chr, chromosome.
