# Supplementary material for: Ambient Temperature is A Strong Selective Factor Influencing Human Development and Immunity
Source: Genomics Proteomics Bioinformatics. 2020 Aug 19;18(5):489–500. doi: 10.1016/j.gpb.2019.11.009 (PMC8377383; doi:10.1016/j.gpb.2019.11.009)
Supplement: Supplementary Table S3 [file mmc3.doc]

**Table S3** **SD-associated SNPs at the suggestive 1×10^-5^ level**

| **Name** | **Rank** | **Chr** | **Gene symbol** | **Location** | ***r*** | ***P*** |
| --- | --- | --- | --- | --- | --- | --- |
| rs25887 | 1 | 5 | *CSF2* | flanking_3UTR | 0.9465 | 3.17×10^-6^ |

*Note*: SD, sunshine duration. Chr, chromosome.
