# Supplementary material for: Ambient Temperature is A Strong Selective Factor Influencing Human Development and Immunity
Source: Genomics Proteomics Bioinformatics. 2020 Aug 19;18(5):489–500. doi: 10.1016/j.gpb.2019.11.009 (PMC8377383; doi:10.1016/j.gpb.2019.11.009)
Supplement: Supplementary Table S4 [file mmc4.doc]

**Table S4** **Pearson bivariate correlation results of the five genome-widely CAT-associated SNPs in Chinese populations**

| **Population** | **rs13729C**  **(*ULBP3*)** | **rs1107877T**  **(*KRT31*)** | **rs12626864T**  **(*LINC00112*)** | **rs9825563A**  **(*DRD3*)** | **rs1444041T**  **(*NTNG1*)** | **CAT (℃)** |  | **N** |
| --- | --- | --- | --- | --- | --- | --- | --- | --- |
| *Dai* | 0.16 | 0.18 | 0.14 | 0.71 | 0.23 | 18.40 |  | 70 |
| *Chinese Russian* | 0.24 | 0.21 | 0.31 | 0.81 | 0.33 | 7.95 |  | 59 |
| Guizhou | 0.19 | 0.15 | 0.31 | 0.64 | 0.35 | 14.05 |  | 51 |
| Henan | 0.21 | 0.13 | 0.30 | 0.70 | 0.37 | 14.22 |  | 94 |
| Liaoning | 0.22 | 0.17 | 0.27 | 0.75 | 0.33 | 8.94 |  | 161 |
| *Miao-GZ* | 0.14 | 0.10 | 0.36 | 0.73 | 0.15 | 15.40 |  | 71 |
| *Miao-HN* | 0.14 | 0.15 | 0.31 | 0.81 | 0.32 | 16.50 |  | 49 |
| Zhejiang | 0.26 | 0.09 | 0.30 | 0.70 | 0.22 | 16.45 |  | 101 |
| Shandong | 0.24 | 0.18 | 0.29 | 0.68 | 0.35 | 13.10 |  | 146 |
| Sichuan | 0.25 | 0.14 | 0.31 | 0.72 | 0.36 | 16.10 |  | 170 |
| Hubei | 0.15 | 0.17 | 0.36 | 0.76 | 0.34 | 16.60 |  | 61 |
| *Zhuang-GX* | 0.12 | 0.20 | 0.23 | 0.69 | 0.22 | 21.63 |  | 145 |
| *Zhunag-YN* | 0.12 | 0.20 | 0.25 | 0.70 | 0.27 | 18.70 |  | 30 |
| Xinjiang | 0.19 | 0.12 | 0.28 | 0.78 | 0.37 | 9.00 |  | 27 |
| Guangdong | 0.16 | 0.10 | 0.25 | 0.70 | 0.30 | 22.50 |  | 160 |
| *r* | –0.555 | –0.081 | –0.359 | –0.447 | –0.471 |  |  |  |
| *P* | 0.032 | 0.775 | 0.188 | 0.095 | 0.076 |  |  |  |

*Note*: Italic font represents ethnic minorities. CAT, climatic ambient temperature. N indicates the sample size.
