# Supplementary material for: Ambient Temperature is A Strong Selective Factor Influencing Human Development and Immunity
Source: Genomics Proteomics Bioinformatics. 2020 Aug 19;18(5):489–500. doi: 10.1016/j.gpb.2019.11.009 (PMC8377383; doi:10.1016/j.gpb.2019.11.009)
Supplement: Supplementary Table S5 [file mmc5.doc]

**Table S5** **Pearson bivariate correlation results of the five genome-widely CAT-associated SNPs in HGDP-CEPH populations**

| **Population** | **rs12202737T**  **(*ULBP3*)** | **rs13729C**  **(*ULBP3*)** | **rs1107877T**  **(*KRT31*)** | **rs12626864T**  **(*LINC00112*)** | **rs9825563A**  **(*DRD3*)** | **rs11185115A**  **(*NTNG1*)** | **CAT (℃)** | **N** |
| --- | --- | --- | --- | --- | --- | --- | --- | --- |
| Chinese | 0.28 | 0.29 | 0.21 | 0.29 | 0.77 | 0.30 | 10.91 | 178 |
| French | 0.25 | 0.25 | 0.14 | 0.18 | 0.69 | 0.29 | 14.01 | 52 |
| Israeli | 0.18 | 0.18 | 0.12 | 0.18 | 0.68 | 0.25 | 17.63 | 134 |
| Italian | 0.23 | 0.23 | 0.16 | 0.24 | 0.70 | 0.28 | 14.51 | 49 |
| Japanese | 0.23 | 0.23 | 0.23 | 0.29 | 0.75 | 0.29 | 11.93 | 29 |
| Kenyan | 0.00 | 0.00 | 0.00 | 0.00 | 0.59 | 0.23 | 29.34 | 11 |
| Nigerian | 0.07 | 0.07 | 0.00 | 0.10 | 0.55 | 0.21 | 27.03 | 22 |
| Pakistani | 0.23 | 0.24 | 0.16 | 0.18 | 0.71 | 0.28 | 18.37 | 192 |
| Russian | 0.30 | 0.30 | 0.26 | 0.27 | 0.79 | 0.32 | 8.08 | 42 |
| Senegal | 0.02 | 0.02 | 0.00 | 0.02 | 0.57 | 0.18 | 28.09 | 22 |
| Siberian | 0.58 | 0.58 | 0.50 | 0.56 | 0.96 | 0.44 | -9.97 | 25 |
| South African | 0.13 | 0.13 | 0.06 | 0.13 | 0.63 | 0.25 | 18.99 | 8 |
| *r* | –0.985 | –0.984 | –0.983 | –0.982 | –0.977 | –0.976 |  |  |
| *P* | 6.73×10^-9^ | 8.37×10^-9^ | 1.01×10^-8^ | 1.66×10^-8^ | 5.26×10^-8^ | 6.26×10^-8^ |  |  |

*Note*: CAT, climatic ambient temperature. N indicates the sample size.
