# Supplementary material for: Ambient Temperature is A Strong Selective Factor Influencing Human Development and Immunity
Source: Genomics Proteomics Bioinformatics. 2020 Aug 19;18(5):489–500. doi: 10.1016/j.gpb.2019.11.009 (PMC8377383; doi:10.1016/j.gpb.2019.11.009)
Supplement: Supplementary Table S6 [file mmc6.doc]

**Table S6** **Functional impact of the CAT genome-wide significant signals**

| **SNP** | **Rank** | **Chr** | **Gene symbol** | **Location** | **Correlated**  **gene/motif** | **Functional effect** | **Tissue** |
| --- | --- | --- | --- | --- | --- | --- | --- |
| rs12202737 | 1 | 6 | *ULBP3* | intron | *ULBP3* | cis-eQTL | Adrenal gland  Cells - transformed fibroblasts  Thyroid |
|  |  |  |  |  | *RAET1L* | cis-eQTL | Esophagus - Mucosa |
|  |  |  |  |  | ERalpha-a_disc2 | Regulatory motifs altered |  |
|  |  |  |  |  | RFX5_known2 |  |
|  |  |  |  |  | Sin3Ak-20_disc3 |  |
| rs13729 | 2 | 6 | *ULBP3* | flanking_3UTR | *ULBP3* | cis-eQTL | Adrenal Gland/Thyroid/Cells - Transformed fibroblasts |
|  |  |  |  |  | *RAET1L* | cis-eQTL | Esophagus - Mucosa |
|  |  |  |  |  | AP-1_disc1 | Regulatory motifs altered |  |
|  |  |  |  |  | ATF3_known8 |  |
|  |  |  |  |  | Cphx |  |
|  |  |  |  |  | Jundm2 |  |
|  |  |  |  |  | TATA_disc2 |  |
| rs1107877 | 3 | 17 | *KRT31* | flanking_3UTR | *KRT32* | cis-eQTL | Testis |
|  |  |  |  |  | *KRTAP3-2* | cis-eQTL | Testis |
|  |  |  |  |  | HDAC2_disc5 | Regulatory motifs altered |  |
|  |  |  |  |  | Ik-2_3 |
|  |  |  |  |  | LRH1 |
|  |  |  |  |  | NF-AT |
|  |  |  |  |  | Pax-1 |
| rs12626864 | 4 | 21 | *LINC00112* | flanking_5UTR | Arid5a | Regulatory motifs altered |  |
|  |  |  |  |  | Ets_disc7 |  |
|  |  |  |  |  | Maf_known3 |  |
|  |  |  |  |  | SRF_known4 |  |
| rs9825563 | 5 | 3 | *DRD3* | flanking_5UTR | DMRT2 | Regulatory motifs altered |  |
|  |  |  |  |  | VDR_2 |  |
|  |  |  |  |  | VDR_4 |  |
| rs11185115 | 6 | 1 | *NTNG1* | intron | - | - |  |

*Note*: CAT, climatic ambient temperature. Chr, chromosome.
