# Supplementary material for: Ambient Temperature is A Strong Selective Factor Influencing Human Development and Immunity
Source: Genomics Proteomics Bioinformatics. 2020 Aug 19;18(5):489–500. doi: 10.1016/j.gpb.2019.11.009 (PMC8377383; doi:10.1016/j.gpb.2019.11.009)
Supplement: Supplementary Table S7 [file mmc7.doc]

**Table S7** **Functional impact of the UVR genome-wide significant signals**

| **SNP** | **Rank** | **Chr** | **Gene symbol** | **Location** | **Correlated gene/motif** | **Functional effect** | **Tissue** |
| --- | --- | --- | --- | --- | --- | --- | --- |
| rs7531583 | 1 | 1 | *NADK* | intron | *CALML6* | cis-eQTL | Adipose-Subcutaneous  Adrenal Gland  Artery – Aorta  Nerve - Tibial  Skin - Not Sun Exposed (Suprapubic)  Skin - Sun Exposed (Lower leg)  Testis  Thyroid |
|  |  |  |  |  | *CDK11A* | cis-eQTL | Lung |
|  |  |  |  |  | *MMP23A* | cis-eQTL | Pancreas |
|  |  |  |  |  | *NADK* | cis-eQTL | Adipose – Subcutaneous  Artery – Aorta  Heart - Left Ventricle  Muscle - Skeletal  Nerve – Tibial  Thyroid |
|  |  |  |  |  | *RP1-140A9.1* | cis-eQTL | Adipose-Subcutaneous  Adipose - Visceral (Omentum)  Adrenal Gland  Brain - Amygdala  Brain - Cerebellar Hemisphere  Brain Cortex  Brain - Frontal Cortex (BA9)  Brain - Hippocampus  Brain - Nucleus accumbens (basal ganglia)  Brain - Putamen (basal ganglia)  Breast - Mammary Tissue  Cells - Transformed fibroblasts  Colon - Sigmoid  Colon - Transverse  Esophagus - Mucosa  Heart - Atrial Appendage  Heart - Left Ventricle  Lung  Muscle - Skeletal  Nerve - Tibial  Pancreas  Pituitary  Prostate |

| rs7531583 | 1 | 1 | *NADK* | intron | *RP1-140A9.1* | cis-eQTL | Skin - Not Sun Exposed (Suprapubic)  Skin - Sun Exposed (Lower leg)  Stomach  Testis  Thyroid  Whole Blood | |
| --- | --- | --- | --- | --- | --- | --- | --- | --- |
|  |  |  |  |  | *TMEM52* | cis-eQTL | Skin - Sun Exposed (Lower leg) | |
|  |  |  |  |  | Foxd3 | Regulatory motifs altered |  | |
|  |  |  |  |  | Foxj1_1 |  |  |
|  |  |  |  |  | Hmx_2 |  |  |
|  |  |  |  |  | Pou3f2_4 |  |  |
|  |  |  |  |  | Pou3f4 |  |  |
|  |  |  |  |  | Sox_3 |  |  |
| rs1586360 | 2 | 7 | *IGFBP3* | flanking_5UTR | *IGFBP1* | cis-eQTL | Artery - Coronary | |
|  |  |  |  |  | Irf_known7 | Regulatory motifs altered |  | |
|  |  |  |  |  | Mef2_known5 |  | |
|  |  |  |  |  | OTX |  | |
|  |  |  |  |  | Obox6 |  | |

*Note*: UVR, ultraviolet radiation. Chr, chromosome.
