# Supplementary material for: Ambient Temperature is A Strong Selective Factor Influencing Human Development and Immunity
Source: Genomics Proteomics Bioinformatics. 2020 Aug 19;18(5):489–500. doi: 10.1016/j.gpb.2019.11.009 (PMC8377383; doi:10.1016/j.gpb.2019.11.009)
Supplement: Supplementary Table S8 [file mmc8.doc]

**Table S8 GO annotations for the** **solar radiation-correlated genes** (DAVID)

| **Category** | **Bonferroni *P* value** | **Percentage of related genes (%)** | **Adjusted fold enrichment** | ***χ^2^***  ***P* value** |
| --- | --- | --- | --- | --- |
| **Biological process** |  |  |  |  |
| GO:0048869 cellular developmental process | 3.48×10^-5^ | 17.30 | 1.64 | 3.07×10^-6^ |
| GO:0030154 cell differentiation | 6.98×10^-5^ | 16.63 | 1.64 | 2.51×10^-5^ |
| GO:0048856 anatomical structure development | 1.97×10^-4^ | 22.25 | 1.39 | 1.05×10^-9^ |
| GO:0048731 system development | 5.09×10^-4^ | 20.67 | 1.39 | 2.07×10^-12^ |
| GO:0007399 nervous system development | 1.24×10^-3^ | 11.91 | 1.74 | 3.13×10^-13^ |
| GO:0032502 developmental process | 1.58×10^-3^ | 25.39 | 1.30 | 2.16×10^-4^ |
| GO:0007275 multicellular organismal development | 3.50×10^-3^ | 23.37 | 1.30 | 2.46×10^-9^ |
| GO:0045595 regulation of cell differentiation | 3.60×10^-3^ | 6.97 | 2.26 | 6.50×10^-34^ |
| GO:0050793 regulation of developmental process | 0.02 | 8.09 | 1.93 | 5.24×10^-27^ |
| GO:0009653 anatomical structure morphogenesis | 0.02 | 11.91 | 1.56 | 1.08×10^-5^ |
| GO:0007155 cell adhesion | 0.04 | 8.09 | 1.90 | 1.36×10^-4^ |
| GO:0022610 biological adhesion | 0.04 | 8.09 | 1.89 | 1.36×10^-4^ |
| **Cell Component** |  |  |  |  |
| GO:0005886 plasma membrane | 4.61×10^-3^ | 27.87 | 1.29 | 3.60×10^-8^ |
| GO:0044459 plasma membrane part | 0.01 | 18.20 | 1.46 | 7.49×10^-7^ |
| GO:0016020 membrane | 0.01 | 46.52 | 1.21 | 6.32×10^-6^ |
| GO:0042995 cell projection | 0.03 | 7.64 | 1.77 | 1.19×10^-15^ |
| GO:0044425 membrane part | 0.04 | 42.25 | 1.20 | 6.78×10^-8^ |
| **Molecular Function** |  |  |  |  |
| GO:0005515 protein binding | 7.92×10^-4^ | 53.26 | 1.08 | 4.25×10^-10^ |
| GO:0019198 transmembrane receptor protein phosphatase activity | 0.03 | 1.35 | 10.51 | 2.70×10^-11^ |
| **INTERPRO** |  |  |  |  |
| IPR013098:Immunoglobulin I-set | 6.93×10^-4^ | 3.60 | 4.45 | 3.25×10^-12^ |
| IPR003598:Immunoglobulin subtype 2 | 0.01 | 4.04 | 3.25 | 6.39×10^-9^ |
| IPR003961:Fibronectin, type III | 0.01 | 3.82 | 3.13 | 8.54×10^-8^ |
| **SP_PIR_KEYWORDS** |  |  |  |  |
| Membrane | 2.94×10^-4^ | 42.47 | 1.19 | 1.79×10^-10^ |
| Glycoprotein | 5.61×10^-4^ | 31.46 | 1.22 | 6.26×10^-7^ |
| Signal | 2.05×10^-3^ | 24.72 | 1.32 | 9.47×10^-7^ |
| Cell adhesion | 4.31×10^-3^ | 5.84 | 2.27 | 7.18×10^-7^ |
| **UP_SEQ_FEATURE** |  |  |  |  |
| Domain:Ig-like C2-type 1 | 0.01 | 3.82 | 3.69 | 1.95×10^-10^ |
| Signal peptide | 0.01 | 24.72 | NA | NA |
| Domain:Ig-like C2-type 2 | 0.01 | 3.82 | 3.67 | 1.95×10^-10^ |
| Domain:Ig-like C2-type 3 | 0.03 | 2.92 | 4.10 | 1.11×10^-8^ |
| Glycosylation site:N-linked (GlcNAc...) | 0.04 | 29.21 | 1.35 | 2.53×10^-9^ |

*Note*: Bonferroni *P* values are calculated from the modified one-tail Fisher Exact Test, supplied by the DAVID online analysis. Percentage of related genes is the percentage of each category genes found among all solar radiation-associated genes. Adjusted fold enrichment derives from the ratio of the percentages for each category genes found among solar radiation-correlated genes and all 17,169 genes from the Illumina 650Y platform. Similarly, *χ^2^ P* values are calculated according to the numbers of related genes found among solar radiation-correlated genes and for the 17,169 genes. Here, number and percentage of each GO category for all 17,169 Illumina 650Y platform genes work as null distribution. NA means no such GO annotation terms found in the null distribution; therefore, an adjusted fold enrichment and *χ^2^* *P* value cannot be calculated.
