# Supplementary material for: Ambient Temperature is A Strong Selective Factor Influencing Human Development and Immunity
Source: Genomics Proteomics Bioinformatics. 2020 Aug 19;18(5):489–500. doi: 10.1016/j.gpb.2019.11.009 (PMC8377383; doi:10.1016/j.gpb.2019.11.009)
Supplement: Supplementary Table S9 [file mmc9.doc]

**Table S9** CAT-associated genes overlap with GWAS gene loci

| **Phenotype** | **CAT genes number** | **CEPH ratio** | **GWAS ratio** |
| --- | --- | --- | --- |
| Acne (severe)* | 6 | 21.9 | 16.7 |
| Waist-hip ratio** | 6 | 14.6 | 10.9 |
| Eating disorders (purging via substances)* | 5 | 13.7 | 21.4 |
| Parasitemia in Tripanosoma cruzi seropositivity* | 5 | 11.0 | 9.8 |
| Motion sickness | 7 | 10.2 | 9.4 |
| Obesity (early onset extreme)** | 5 | 10.0 | 9.8 |
| Inflammatory biomarkers* | 5 | 9.1 | 8.1 |
| Allergic rhinitis* | 5 | 7.8 | 6.7 |
| Breast size | 5 | 7.3 | 6.2 |
| Smooth-surface caries | 5 | 7.3 | 5.6 |
| Prostate cancer (gene x gene interaction) | 6 | 6.9 | 5.6 |
| Alcohol dependence* | 5 | 6.8 | 8.1 |
| Immune response to smallpox (secreted IFN-alpha)* | 5 | 6.8 | 7.4 |
| Response to statin therapy | 5 | 6.4 | 6.2 |
| Visceral adipose tissue/subcutaneous adipose tissue ratio | 5 | 6.4 | 5.6 |
| Protein quantitative trait loci | 7 | 6.1 | 6.7 |
| Visceral adipose tissue adjusted for BMI | 5 | 5.8 | 5.2 |
| Bipolar disorder and schizophrenia* | 12 | 5.5 | 4.6 |
| Amyotrophic lateral sclerosis (sporadic) | 11 | 5.5 | 2.9 |
| Major depressive disorder* | 6 | 4.9 | 3.4 |
| Urate levels in obese individuals | 5 | 4.8 | 6.7 |
| Copper levels | 5 | 4.8 | 5.6 |
| Heart rate | 5 | 4.4 | 6.4 |
| Cognitive function | 5 | 4.1 | 3.9 |
| Diisocyanate-induced asthma* | 14 | 4.0 | 3.7 |
| Cognitive performance | 9 | 3.9 | 3.5 |
| Colorectal cancer* | 8 | 3.8 | 3.3 |
| Urate levels in overweight individuals | 5 | 3.4 | 5.5 |
| Obesity | 6 | 3.4 | 2.7 |
| Inflammatory skin disease* | 6 | 2.9 | 3.2 |
| Alzheimer's disease | 5 | 2.8 | 3.6 |
| Prostate cancer* | 10 | 2.4 | 2.3 |
| Inflammatory bowel disease* | 7 | 2.3 | 2.8 |
| Schizophrenia* | 13 | 2.2 | 2.5 |
| Obesity-related traits | 36 | 1.7 | 1.8 |
| Body mass index | 27 | 1.5 | 1.5 |

*Note*: CAT genes number is the number of CAT-associated genes relevant with the GWAS phenotype. Fold enrichment is indicated by CEPH ratio and GWAS ratio. Specifically, CEPH ratio derives from the comparison between the percentages for each phenotype associated gene among 265 CAT-associated genes and all HGDP-CEPH mapped genes. Similarly, GWAS ratio derives from the comparison between the percentages for each phenotype associated gene among 265 CAT-associated genes and all GWAS catalog genes. ** and * indicates the fold enrichment is statistically significant (**, *χ²*; *P* < 0.005. *, *χ²*; *P* < 0.05.). CAT, climatic ambient temperature.
