# Supplementary material for: Ambient Temperature is A Strong Selective Factor Influencing Human Development and Immunity
Source: Genomics Proteomics Bioinformatics. 2020 Aug 19;18(5):489–500. doi: 10.1016/j.gpb.2019.11.009 (PMC8377383; doi:10.1016/j.gpb.2019.11.009)
Supplement: Supplementary Table S10 [file mmc10.doc]

**Table S10** **Fixation of CAT-associated SNPs in different populations**

| **Population** | **Fixed ancestral allele (%)** | **Fixed derived allele (%)** | **Total fixed (%)** |
| --- | --- | --- | --- |
| All African populations | 46 (11.5) | 3 (0.7) | 49 (12.2) |
| Kenyan population | 130 (32.4) | 14 (3.5) | 144 (35.9) |
| Nigerian population | 99 (24.7) | 6 (1.5) | 105 (26.2) |
| Senegal population | 113 (28.2) | 8 (2.0) | 121 (30.2) |
| South African population | 72 (18.0) | 3 (0.7) | 75 (18.7) |
| Siberian population | 2 (0.5) | 1 (0.2) | 3 (0.7) |

*Note*: CAT, climatic ambient temperature.
