# Supplementary material for: Ambient Temperature is A Strong Selective Factor Influencing Human Development and Immunity
Source: Genomics Proteomics Bioinformatics. 2020 Aug 19;18(5):489–500. doi: 10.1016/j.gpb.2019.11.009 (PMC8377383; doi:10.1016/j.gpb.2019.11.009)
Supplement: Supplementary Table S11 [file mmc11.doc]

**Table S11 12 merged African or Euro-Asian human populations (N =** 764)

| **Sampling region** | **Sample size** | **HGDP populations** |
| --- | --- | --- |
| ***Asia*** |  |  |
| China | 178 | Dai, Daur, Han, Hezhe, Lahu, Miaozu, Mongola, Naxi, Oroqen, She, Tu, Tujia, Uygur, Xibo, and Yizu |
| Japan | 29 | Japanese |
| Siberia | 25 | Yakut |
| Pakistan | 192 | Balochi, Brahui, Burusho, Hazara, Kalash, Makrani, Pathan, and Sindhi |
| ***Europe*** |  |  |
| France | 52 | French and French Basque |
| Italy | 49 | Sardinian, Tuscan, and North Italian |
| Russia | 42 | Russian and Adygei |
| ***Middle East*** |  |  |
| Israel | 134 | Druze, Palestinian, and Bedouin |
| ***Subsaharian Africa*** |  |  |
| Kenya | 11 | Bantu N.E. |
| Nigeria | 22 | Yoruba |
| Senegal | 22 | Mandenka |
| South Africa | 8 | Bantu S.E., Bantu S.W. |

*Note*: Six African, Asian, or European populations (Biaka Pygmy, Cambodian, Mbuti Pygmy, Mozabite, Orcadian, and San) are excluded due to lack of specific climatic data.
