# Supplementary material for: Ambient Temperature is A Strong Selective Factor Influencing Human Development and Immunity
Source: Genomics Proteomics Bioinformatics. 2020 Aug 19;18(5):489–500. doi: 10.1016/j.gpb.2019.11.009 (PMC8377383; doi:10.1016/j.gpb.2019.11.009)
Supplement: Supplementary Table S12 [file mmc12.doc]

**Table S12 Pairwise *F*_ST_ for the four continental population**s

| **Populations** | ***F*_ST_** | |
| --- | --- | --- |
|  | **CAT** | **Control** |
| Asian & European | 0.003* | 0.045 |
| European & Middle Eastern | 0.011 | 0.011 |
| Asian & Middle Eastern | 0.021* | 0.048 |
| Sub-Saharan African & Middle Eastern | 0.060* | 0.102 |
| Sub-Saharan African & European | 0.097* | 0.123 |
| Sub-Saharan African & Asian | 0.106 | 0.110 |

*Note*: CAT, climatic ambient temperature. *, unpaired two-tailed Student's *t* test, *P* < 0.001.
