# Supplementary material for: Ambient Temperature is A Strong Selective Factor Influencing Human Development and Immunity
Source: Genomics Proteomics Bioinformatics. 2020 Aug 19;18(5):489–500. doi: 10.1016/j.gpb.2019.11.009 (PMC8377383; doi:10.1016/j.gpb.2019.11.009)
Supplement: Supplementary Table S13 [file mmc13.doc]

**Table S13 Pairwise *F*_ST_** for the three HapMap populations

| **Populations** | ***F*_ST_** | |
| --- | --- | --- |
|  | **CAT** | **Control** |
| ASN & CEU | 0.020* | 0.108 |
| ASN & YRI | 0.151 | 0.148 |
| CEU & YRI | 0.119* | 0.143 |

*Note*: ASN, Asians, includes HapMap CHB and JPT populations. CEU, Caucasians. YRI, Africans. CAT, climatic ambient temperature. *, unpaired two-tailed Student's *t* test, *P* < 0.01.
