# Supplementary material for: Ambient Temperature is A Strong Selective Factor Influencing Human Development and Immunity
Source: Genomics Proteomics Bioinformatics. 2020 Aug 19;18(5):489–500. doi: 10.1016/j.gpb.2019.11.009 (PMC8377383; doi:10.1016/j.gpb.2019.11.009)
Supplement: Supplementary Table S14 [file mmc14.doc]

**Table S14 CAT-associated SNPs with high *F*_ST_ values in HGDP-CEPH populations (Top 10)**

| **No.** | **Name** | **Chr** | **Gene** | **Location** | ***F*_ST_** |
| --- | --- | --- | --- | --- | --- |
| **African *vs*. Middle Eastern populations (mean: 0.060)** | | | | | |
| 1 | rs174546 | 11 | *FADS1* | 3UTR | 0.236 |
| 2 | rs174556 | 11 | *FADS1* | intron | 0.217 |
| 3 | rs12525101 | 6 | *FARS2* | intron | 0.207 |
| 4 | rs2825319 | 21 | *PRSS7* | flanking_5UTR | 0.199 |
| 5 | rs535765 | 19 | *ZNRF4* | flanking_5UTR | 0.197 |
| 6 | rs2968800 | 2 | *CLHC1* | flanking_3UTR | 0.196 |
| 7 | rs1350380 | 16 | *GPR139* | flanking_3UTR | 0.195 |
| 8 | rs234460 | 14 | *VRK1* | flanking_3UTR | 0.193 |
| 9 | rs1342517 | 15 | *MCTP2* | flanking_5UTR | 0.172 |
| 10 | rs12613347 | 2 | *CASP10* | intron | 0.170 |
| **African *vs*. Asian populations (mean: 0.106)** | | | | | |
| 1 | rs1342517 | 15 | *MCTP2* | flanking_5UTR | 0.346 |
| 2 | rs2735202 | 11 | *ZBTB16* | intron | 0.309 |
| 3 | rs12702671 | 7 | *GLCCI1* | flanking_5UTR | 0.281 |
| 4 | rs1957015 | 14 | *AKAP6* | intron | 0.253 |
| 5 | rs174546 | 11 | *FADS1* | 3UTR | 0.247 |
| 6 | rs16823913 | 2 | *CCL20* | flanking_5UTR | 0.247 |
| 7 | rs10871579 | 18 | *LINC00305* | flanking_5UTR | 0.238 |
| 8 | rs13203409 | 6 | *LOC441179* | flanking_3UTR | 0.235 |
| 9 | rs2968800 | 2 | *CLHC1* | flanking_3UTR | 0.233 |
| 10 | rs2825319 | 21 | *PRSS7* | flanking_5UTR | 0.231 |
| **Middle Eastern *vs*. Asian populations (mean: 0.021)** | | | | | |
| 1 | rs4358672 | chr6 | *LOC441179* | flanking_3UTR | 0.145 |
| 2 | rs12702671 | chr7 | *GLCCI1* | flanking_5UTR | 0.121 |
| 3 | rs6545433 | chr2 | *SPTBN1* | intron | 0.099 |
| 4 | rs1250550 | chr10 | *ZMIZ1* | intron | 0.096 |
| 5 | rs10953303 | chr7 | *ZAN* | coding | 0.095 |
| 6 | rs2735202 | chr11 | *ZBTB16* | intron | 0.094 |
| 7 | rs4144407 | chr18 | *SYT4* | flanking_5UTR | 0.093 |
| 8 | rs16869739 | chr4 | *SLIT2* | intron | 0.091 |
| 9 | rs1939957 | chr11 | *OPCML* | intron | 0.089 |
| 10 | rs1342517 | chr15 | *MCTP2* | flanking_5UTR | 0.087 |
| **African *vs*. European populations (mean: 0.097)** | | | | | |
| 1 | rs1342517 | chr15 | *MCTP2* | flanking_5UTR | 0.277 |
| 2 | rs11780158 | chr8 | *CCDC25* | flanking_3UTR | 0.264 |
| 3 | rs6545433 | chr2 | *SPTBN1* | intron | 0.259 |
| 4 | rs762308 | chr3 | *ITGA9* | intron | 0.227 |
| 5 | rs2654189 | chr17 | *SLC2A4* | flanking_5UTR | 0.227 |
| 6 | rs12879663 | chr14 | *MARK3* | intron | 0.225 |
| 7 | rs13203409 | chr6 | *LOC441179* | flanking_3UTR | 0.225 |
| 8 | rs495296 | chr1 | *KCNK2* | flanking_5UTR | 0.225 |
| 9 | rs2825319 | chr21 | *PRSS7* | flanking_5UTR | 0.219 |
| 10 | rs12702671 | chr7 | *GLCCI1* | flanking_5UTR | 0.218 |
| **Middle Eastern *vs*. European populations (mean: 0.012)** | | | | | |
| 1 | rs6545433 | chr2 | *SPTBN1* | intron | 0.109 |
| 2 | rs2269679 | chr1 | *ADCY10* | intron | 0.093 |
| 3 | rs2654189 | chr17 | *SLC2A4* | flanking_5UTR | 0.077 |
| 4 | rs4144407 | chr18 | *SYT4* | flanking_5UTR | 0.077 |
| 5 | rs4358672 | chr6 | *LOC441179* | flanking_3UTR | 0.073 |
| 6 | rs2052037 | chr19 | *UQCRFS1* | flanking_3UTR | 0.071 |
| 7 | rs6438101 | chr3 | *CD200R1L* | flanking_3UTR | 0.066 |
| 8 | rs1939957 | chr11 | *OPCML* | intron | 0.066 |
| 9 | rs495296 | chr1 | *KCNK2* | flanking_5UTR | 0.057 |
| 10 | rs591269 | chr6 | *CITED2* | flanking_5UTR | 0.057 |
| **Asian *vs*. European populations (mean: 0.004)** | | | | | |
| 1 | rs16869739 | chr4 | *SLIT2* | intron | 0.038 |
| 2 | rs2735202 | chr11 | *ZBTB16* | intron | 0.037 |
| 3 | rs34569 | chr5 | *EFNA5* | flanking_3UTR | 0.034 |
| 4 | rs12056093 | chr7 | *AUTS2* | intron | 0.028 |
| 5 | rs199635 | chr6 | *C6orf155* | flanking_5UTR | 0.027 |
| 6 | rs1232783 | chr20 | *PLCB1* | intron | 0.026 |
| 7 | rs10871579 | chr18 | *LINC00305* | flanking_5UTR | 0.026 |
| 8 | rs174556 | chr11 | *FADS1* | intron | 0.025 |
| 9 | rs1535 | chr11 | *FADS2* | intron | 0.023 |
| 10 | rs2022345 | chr6 | *DTNBP1* | flanking_5UTR | 0.023 |

*Note*: CAT, climatic ambient temperature. Chr, chromosome.
