# Supplementary material for: Ambient Temperature is A Strong Selective Factor Influencing Human Development and Immunity
Source: Genomics Proteomics Bioinformatics. 2020 Aug 19;18(5):489–500. doi: 10.1016/j.gpb.2019.11.009 (PMC8377383; doi:10.1016/j.gpb.2019.11.009)
Supplement: Supplementary Table S15 [file mmc15.doc]

**Table S15** **SNPs with high iHS scores in HapMap populations (|iHS| ≥ 2)**

| **Population** | **SNP** | **Gene** | **iHS** |
| --- | --- | --- | --- |
| ASN | rs1003260 | *RIMS1* | 2.386 |
|  | rs11629363 | *DACT1* | –2.203 |
|  | rs1428479 | *CD180* | 2.815 |
|  | rs156769 | *ANXA1* | 2.120 |
|  | **rs16823913** | ***CCL20*** | **2.843** |
|  | rs1727668 | *LINC00472* | –2.065 |
|  | ***rs174556*** | ***FADS1*** | **2.383** |
|  | rs1931708 | *DOCK1* | –2.192 |
|  | rs2968800 | *CLHC1* | 2.005 |
|  | rs3767141 | *HSPG2* | 2.242 |
|  | rs4999155 | *SPATA31D2P* | 2.478 |
|  | **rs657672** | ***CACNB1*** | **2.053** |
|  | rs7502935 | *TIMP2* | –2.305 |
|  | rs8094855 | *CBLN2* | –2.204 |
|  | rs898716 | *FRMD4A* | –2.014 |
| CEU | rs10903595 | *ADARB2* | –2.221 |
|  | rs138705 | *UNC84B* | 2.083 |
|  | rs1520 | *KIF6* | –2.261 |
|  | **rs16823913** | ***CCL20*** | **3.335** |
|  | rs16960758 | *SLC12A1* | 2.187 |
|  | rs2269679 | *ADCY10* | 2.016 |
|  | rs32018 | *CD180* | 2.045 |
|  | rs556925 | *MBNL1* | –2.033 |
|  | **rs657672** | ***CACNB1*** | **2.015** |
|  | rs9402494 | *EYA4* | –2.119 |
| YRI | rs10764 | *CLASP2* | 2.835 |
|  | rs10943606 | *PHIP* | –2.429 |
|  | ***rs174556*** | ***FADS1*** | **2.149** |
|  | rs1957015 | *AKAP6* | 2.276 |
|  | rs2076962 | *FBXO31* | 2.187 |
|  | rs439022 | *SLITRK5* | 2.009 |
|  | rs4806741 | *LILRA3* | 2.528 |
|  | rs767739 | *TMC2* | –2.231 |

*Note*: ASN, Asians, includes HapMap CHB and JPT populations. CEU, Caucasians. YRI, Africans. Bold face alone indicates the SNP is shared between ASN and CEU populations, while bold and italic face indicates the SNP is shared between ASN and YRI populations. For each SNP, the iHS score was retrieved from Haplotter (http://haplotter.uchicago.edu/).
