# Supplementary material for: Ambient Temperature is A Strong Selective Factor Influencing Human Development and Immunity
Source: Genomics Proteomics Bioinformatics. 2020 Aug 19;18(5):489–500. doi: 10.1016/j.gpb.2019.11.009 (PMC8377383; doi:10.1016/j.gpb.2019.11.009)
Supplement: Supplementary Table S16 [file mmc16.doc]

**Table S16 SNPs with high iHS scores in HGDP-CEPH populations (|iHS| ≥ 2)**

| **Population** | **SNP** | **Gene** |
| --- | --- | --- |
| African population | rs1020684 | *SLITRK5* |
|  | rs394028 | *SLITRK5* |
|  | rs439022 | *SLITRK5* |
|  | rs7328476 | *SLITRK5* |
|  | rs17461918 | *MAGI3* |
|  | rs7514649 | *MAGI3* |
|  | rs7647008 | *CLASP2* |
| East Asian population | rs10942089 | *CDH10* |
| European population | rs1200610 | *SWT1* |
|  | rs16960758 | *SLC12A1* |
|  | rs11208527 | *RAVER2* |
| Middle Eastern population | rs10942089 | *CDH10* |
|  | rs16960758 | *SLC12A1* |
|  | rs11208527 | *RAVER2* |
| South Asian population | rs10942089 | *CDH10* |
|  | rs16960758 | *SLC12A1* |

*Note*: African population includes Bantu Kenya, Bantu South Africa, Biaka Pygmy, Mandenka, Mbuti Pygmy, San, and Yoruba. East Asian population includes Cambodian, Dai, Daur, Han, Hezhen, Japanese, Lahu, Miao, Mogola, Naxi, Oroqen, She, Tu, Tujia, Xibo, Yakut, and Yi. European population includes Adygei, Basque, French, Italian, Orcadian, Russian, Sardinian, and Tuscan. Middle Eastern population includes Bedouin, Druze, Mozabite, and Palestinian. South Asian population includes Balochi, Brahui, Burusho, Hazara, Kalash, Makrani, Pathan, Sindhi, and Uygur. For each SNP, the iHS score was retrieved from the HGDP Selection Browser (http://hgdp.uchicago.edu/cgi-bin/gbrowse/HGDP/).
