# Supplementary material for: Ambient Temperature is A Strong Selective Factor Influencing Human Development and Immunity
Source: Genomics Proteomics Bioinformatics. 2020 Aug 19;18(5):489–500. doi: 10.1016/j.gpb.2019.11.009 (PMC8377383; doi:10.1016/j.gpb.2019.11.009)
Supplement: Supplementary Table S17 [file mmc17.doc]

**Table S17** **Replicating Chinese human populations (N = 1395)**

| **Sampling province** | **Ethnic group** | **Sample size** |
| --- | --- | --- |
| Guangdong | Han | 160 |
| Guizhou | Han | 51 |
| Henan | Han | 94 |
| Liaoning | Han | 161 |
| Zhejiang | Han | 101 |
| Shandong | Han | 146 |
| Sichuan | Han | 170 |
| Hubei | Han | 61 |
| Xinjiang | Han | 27 |
| Guizhou (gz) | Miao | 71 |
| Hunan (hn) | Miao | 49 |
| Yunnan (yn) | Zhuang | 30 |
| Guangxi (gx) | Zhuang | 145 |
| Yunnan (yn) | Dai | 70 |
| Inner Mongolia Autonomous Region | Chinese Russian | 59 |
