# Supplementary material for: Ambient Temperature is A Strong Selective Factor Influencing Human Development and Immunity
Source: Genomics Proteomics Bioinformatics. 2020 Aug 19;18(5):489–500. doi: 10.1016/j.gpb.2019.11.009 (PMC8377383; doi:10.1016/j.gpb.2019.11.009)
Supplement: Supplementary Table S18 [file mmc18.doc]

**Table S18** **Climatological information for HGDP-CEPH populations**

| **Population** | **CAT (℃)** | **SD (h)** | **UVR yearly (0.01 MJ/m^2^)** |
| --- | --- | --- | --- |
| China | 10.91 | 6.39 | 54191 |
| France | 14.01 | 6.28 | 31716 |
| Israel | 17.63 | 9.18 | 66259 |
| Italy | 14.51 | 6.22 | 37793 |
| Japan | 11.93 | 5.04 | 40765 |
| Kenya | 29.34 | 8.53 | 100450 |
| Nigeria | 27.03 | 7.01 | 84810 |
| Pakistan | 18.37 | 8.31 | 75249 |
| Russia | 8.08 | 5.45 | 25626 |
| Senegal | 28.09 | 8.13 | 93997 |
| Siberia | –9.97 | 6.09 | 16114 |
| South Africa | 18.99 | 8.34 | 80834 |

*Note*: CAT, climatic ambient temperature. SD, sunshine duration. UVR, ultraviolet radiation.
