# Supplementary material for: Ambient Temperature is A Strong Selective Factor Influencing Human Development and Immunity
Source: Genomics Proteomics Bioinformatics. 2020 Aug 19;18(5):489–500. doi: 10.1016/j.gpb.2019.11.009 (PMC8377383; doi:10.1016/j.gpb.2019.11.009)
Supplement: Supplementary Table S19 [file mmc19.doc]

**Table S19** **Climatological information for Chinese populations**

| **Population** | **Max** $\overline{T}$ | **Min** $\overline{T}$ | $\overline{T}$ ***r*ange** | **extreme**  **max** $\overline{T}$ | **extreme**  **min** $\overline{T}$ | **CAT**  **(℃)** | **SD**  **(h)** | **UVR**  **(0.01 MJ/m^2^)** |
| --- | --- | --- | --- | --- | --- | --- | --- | --- |
| Guizhou | 25.43 | 13.80 | 102.00 | 35.533 | –1.733 | 14.05 | 8.52 | 18.40 |
| Henan | 14.45 | 2.35 | 341.00 | 39.850 | –34.400 | 14.22 | 9.72 | 7.95 |
| Liaoning | 18.85 | 10.85 | 173.50 | 35.850 | –8.550 | 8.94 | 7.68 | 14.05 |
| Zhejiang | 19.90 | 9.52 | 263.60 | 41.000 | –17.740 | 16.45 | 8.40 | 14.22 |
| Shandong | 14.38 | 4.19 | 332.50 | 37.675 | –28.050 | 13.10 | 9.00 | 8.94 |
| *Miao-GZ* | 16.30 | 2.50 | 319.00 | 39.200 | –36.000 | 15.40 | 8.76 | 15.40 |
| *Miao-HN* | 26.20 | 19.60 | 131.00 | 37.500 | 2.200 | 16.50 | 9.72 | 16.50 |
| Sichuan | 19.80 | 12.30 | 188.00 | 35.100 | –7.300 | 16.10 | 8.40 | 16.45 |
| Hubei | 21.10 | 13.20 | 223.00 | 39.100 | –11.500 | 16.60 | 8.16 | 13.10 |
| Xinjiang | 20.50 | 13.40 | 227.00 | 39.250 | –7.050 | 9.00 | 8.64 | 16.10 |
| Guangdong | 18.30 | 8.70 | 277.50 | 38.900 | –17.617 | 22.50 | 9.12 | 16.60 |
| *Zhuang-GX* | 20.30 | 13.10 | 196.00 | 36.700 | –5.900 | 21.63 | 8.28 | 21.63 |
| *Zhunag-YN* | 21.10 | 13.10 | 250.00 | 39.300 | –18.100 | 18.70 | 8.40 | 18.70 |
| *Russian* | 24.60 | 14.60 | 109.00 | 35.900 | –3.900 | 7.95 | 8.40 | 22.50 |
| *Dai* | 26.48 | 18.43 | 154.75 | 40.125 | –0.500 | 18.40 | 9.36 | 9.00 |

*Note*: $\overline{T}$ means annual average CAT. CAT, climatic ambient temperature. Max/Min means maximum/minimum.
